# Bioprospection of Phytotoxic Plant-Derived Eudesmanolides and Guaianolides for the Control of *Amaranthus viridis*, *Echinochloa crus-galli*, and *Lolium perenne* Weeds

**DOI:** 10.1021/acs.jafc.3c06901

**Published:** 2024-01-11

**Authors:** Jesús G. Zorrilla, David M. Cárdenas, Carlos Rial, José M.G. Molinillo, Rosa M. Varela, Marco Masi, Francisco A. Macías

**Affiliations:** † Department of Chemical Sciences, University of Naples Federico II, Complesso Universitario Monte S. Angelo, Via Cinthia 4, 80126 Naples, Italy; ‡ Allelopathy Group, Department of Organic Chemistry, Facultad de Ciencias, Institute of Biomolecules (INBIO), University of Cadiz, C/Avenida República Saharaui, 7, 11510 Puerto Real, Spain

**Keywords:** phytotoxicity studies, weed control, herbicide development models, bioassays, sesquiterpene lactones, structure−activity relationships

## Abstract

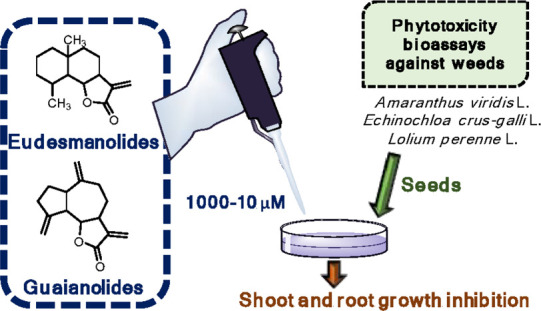

The phytotoxicities of a selection of eudesmanolides and guaianolides, including natural products and new derivatives obtained by semisynthesis from plant-isolated sesquiterpene lactones, were evaluated in bioassays against three weeds of concern in agriculture (*Amaranthus viridis* L., *Echinochloa crus-galli* L., and *Lolium perenne* L.). Both eudesmanolides and guaianolides were active against the root and shoot growth of all the species, with the eudesmanolides generally showing improved activities. The IC_50_ values obtained for the herbicide employed as positive control (on root and shoot growth, respectively, *A. viridis*: 27.8 and 85.7 μM; *E. crus-galli*: 167.5 and 288.2 μM; *L. perenne*: 99.1 and 571.4 μM) were improved in most of the cases. Structure–activity relationships were discussed, finding that hydroxylation of the A-ring and C-13 as well as the position, number, and orientation of the hydroxyl groups and the presence of an unsaturated carbonyl group can significantly influence the level of phytotoxicity. γ-Cyclocostunolide was the most active compound in the series, followed by others such as dehydrozaluzanin C and α-cyclocostunolide (outstanding their IC_50_ values on *A. viridis*)—natural products that can therefore be suggested as models for herbicide development if further research indicates effectiveness on a larger scale and environmental safety in ecotoxicological assessments.

## 1 Introduction

Numerous studies on natural products have focused on the search for phytotoxic chemicals to develop effective herbicides that, being based on metabolites produced by organisms that cohabit in ecosystems, minimize the environmental impact and thus contribute to a more sustainable agronomy. Bioherbicides were introduced into the market in the 1980s, and several new examples have been registered since then.^1^ Progress in this complex area and the discovery and study of new phytotoxic metabolites are ongoing,^2−4^ while there are natural sources that are yet to be studied in depth. Progress is accentuated by the need to exploit compounds based on natural products due to restrictions on agricultural practices that are coming into force.^5^ The market presence of herbicides based on natural products is still modest in comparison with fungicides and insecticides.^6^ Including semisynthetic derivatives and mimics, natural products would cover 17% of crop protection compounds.^7^ This gradual growth is supported by the valuable advantages that bioherbicides possess when compared to classical herbicides, which include alternative modes of action, lower prevalence in soils, activity at lower concentrations, and greater selectivity. However, bioherbicides have disadvantages and limitations, such as the frequent low yield obtained in the isolation of natural products and the complex structures they generally have, which makes them challenging to obtain by total synthetic procedures. One solution is the use of efficient synthetic strategies through semisynthesis from isolated compounds. Furthermore, synthesis allows accessibility to new molecules with enhanced biological activities,^8,9^ for example, by the introduction of functional groups that directly provoke phytotoxicity or by the modification of physicochemical properties, like solubility, leading to better access to the target sites.^10,11^

**Figure 1:**
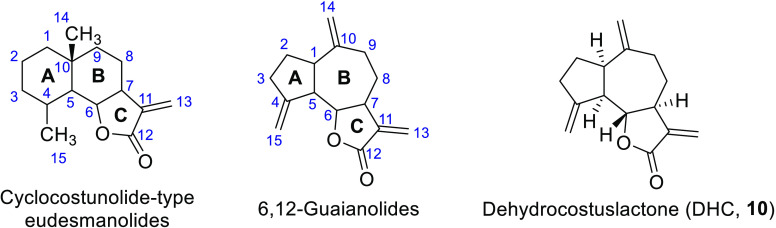
General structures of the compounds studied and that of the guaianolide dehydrocostuslactone (**10**).

Sesquiterpene lactones are natural products that contain a lactone ring strongly linked to their bioactivity, and they also play a physiological role in plants.^12^ Some of the most interesting subgroups are eudesmanolides and guaianolides,^13−15^ although guaianolides have been studied in greater depth than eudesmanolides. Phytotoxicity has been described for both subgroups. Cyclocostunolide-type eudesmanolides (Figure 1) bearing a hydroxyl group at C-1 are active against *Amaranthus viridis* L. and *Echinochloa crus-galli* L. weeds.^16^ A natural substituted guaianolide showed phytotoxicity against *Lolium perenne* L.^17,18^ Moreover, hydroxylation at C-13 of β-cyclocostunolide and the guaianolide dehydrocostuslactone (commonly abbreviated as DHC, **10**, Figure 1) led to phytotoxic derivatives that inhibited the growth of wheat coleoptiles,^19^ although an evaluation against specific weeds was not performed. As a consequence, there is interest in carrying out more detailed phytotoxicity evaluations on sesquiterpene lactones against weeds of these types. The hydroxylated derivatives are promising compounds to explore, bearing in mind the aforementioned background together with other references that highlight the interest of hydroxylated derivatives in the agronomic field,^20,21^ in which it should be noted that hydroxylated derivatives show different behaviors in comparison with their parent compounds.^22^

**Figure 2:**
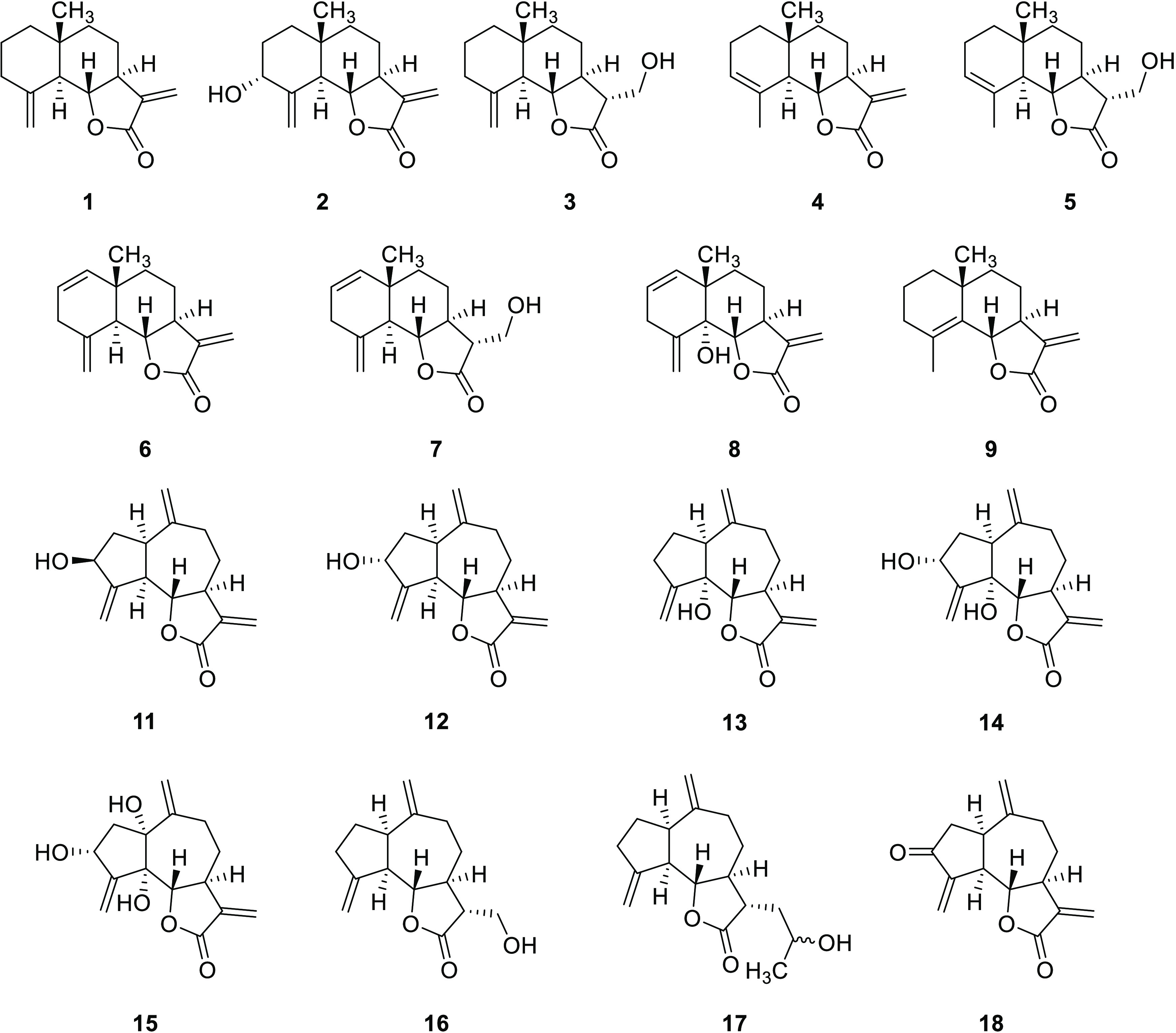
Eudesmanolides (**1**–**9**) and guaianolides (**11**–**18**) tested in the phytotoxicity bioassays: βcyclocostunolide (**1**), 3α-hydroxy-β-cyclocostunolide (**2**), 11α-hydroxymethyl-β-cyclocostunolide (**3**), αcyclocostunolide (**4**), 11α-hydroxymethyl-α-cyclocostunolide (**5**), 3-deoxybrachylaenolide (**6**), 11αhydroxymethyl-3-deoxybrachylaenolide (**7**), 5α-hydroxy-3-deoxybrachylaenolide (**8**), γ-cyclocostunolide (**9**), zaluzanin C (**11**), isozaluzanin C (**12**), 5α-hydroxydehydrocostuslactone (**13**), 5α-hydroxyisozaluzanin C (**14**), 1α,5α-dihydroxyisozaluzanin C (**15**), 11α-hydroxymethyldehydrocostuslactone (**16**), 11α-(2-hydroxypropyl)dehydrocostuslactone (**17**), and dehydrozaluzanin C (**18**).

The aim of this study is to evaluate the phytotoxicity of a wide range of eudesmanolides and guaianolides and the influence of their oxygenated functional groups against three different weed species in the expectation of identifying structure–activity relationships (SAR). The selection of compounds was based on the findings outlined above and considering semisynthetic strategies as tools to obtain some natural products (like zaluzanin C and isozaluzanin C) and new derivatives by hydroxylation of specific positions. Dehydrozaluzanin C (**18**, Figure 2), a natural guaianolide containing an unsaturated carbonyl function in the A-ring, was also considered given the promising results previously obtained with representatives of monocotyledon and dicotyledon weeds,^23^ the alternative modes of action suggested,^24^ and the improvement in the phytotoxicity against some weeds (including *A. viridis* and *E. crus-galli*) achieved by the introduction of an unsaturated carbonyl function in the A-ring in eudesmanolides.^25^

In this work, 17 compounds (Figure 2) were evaluated in bioassays, including the nonhydroxylated compounds **1**, **4**, **6**, and **9** (the phytotoxicity of DHC, **10**, against the tested weeds was reported previously).^26,27^ The weed species studied belong to harmful species for agriculture worldwide. *A. viridis* (slender amaranth) is a prevalent weed in warm temperature regions and is considered to be a major problem for agriculture as it affects more than 50 crops, causes significant biomass loss, and has a fast infestation;^28,29^*E. crus-galli* (barnyard grass) is one of the most noxious weeds in modern agriculture, and it affects relevant crops like rice, is dispersed worldwide, and has a tendency to grow in harsh climates;^30,31^ and *L. perenne* (ryegrass), the major weed that affects wheat production worldwide.^32^

## 2 Materials and Methods

### 2.1 General Experimental Procedures

The structural characterization of compounds was conducted by monodimensional (^1^H and ^13^C), bidimensional (^1^H–^1^H COSY, ^1^H–^13^C HSQC, and ^1^H–^13^C HMBC), and NOE NMR experiments, along with mass spectrometry, specific rotation measurement, and Fourier-transform infrared spectroscopy (FTIR). NMR spectra were recorded on Agilent spectrometers at 500 MHz using CDCl_3_ (Merck) as solvent and using its residual peak as internal reference (δ 7.26 ppm in ^1^H and δ 77.0 ppm in ^13^C NMR). Exact masses were obtained by ultraperformance liquid chromatography coupled with quadrupole time-of-flight mass spectrometry operating in electrospray ionization mode on a Waters SYNAPT G2 high-resolution mass spectrometer. Mass spectra were recorded in the positive-ion mode in the range *m*/*z* 100–2000 Da, with a mass resolution of 20,000 and an acceleration voltage of 0.7 kV. Optical rotation values were measured in CHCl_3_ on a JASCO P-2000 polarimeter. FTIR spectra were obtained on a PerkinElmer Spectrum Two IR spectrophotometer. Major absorptions are given as wavenumbers in cm^–1^.

The purification of compounds was performed by column chromatography on silica gel (Merck, Geduran Si 60, 0.063–0.200 mm).

### 2.2 Starting Material for the Syntheses of Phytotoxic Compounds

**Figure 3:**
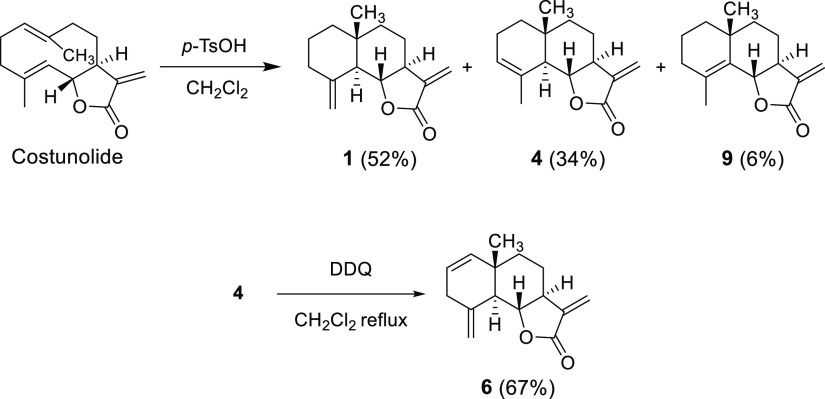
Preparation of β-cyclocostunolide (**1**), α-cyclocostunolide (**4**), γ-cyclocostunolide (**9**), and 3-deoxybrachylaenolide (**6**) from costunolide.

The hydroxylated eudesmanolides were synthesized from compounds **1**, **4**, **6**, or **9**, which were obtained from costunolide (Figure 3), and the hydroxylated guaianolides were synthesized from DHC (**10**), as summarized in Table 1. Both costunolide and DHC were isolated in multigram scale from *Saussurea lappa* root extracts (Pierre Chauvet S.A., France), a rich source of a number of natural products including sesquiterpene lactones.^33^ The extract (50.2 g) was fractioned by column chromatography (13 cm height, 7 cm diameter), and the fractions that contained costunolide and DHC were further purified by column chromatography (10 cm height, 4 cm diameter). The mobile phases were *n*-hexane/ethyl acetate 95:5 (*v*/*v*). Pure costunolide (3.0 g) and DHC (2.6 g) were isolated, and their structures and purities were confirmed by comparison of their ^1^H NMR spectra (Figures S6 and S8.1) and optical rotation values with those reported previously.^34^

**Table 1:** Synthetic Information and Yields Obtained from the Syntheses of Products **1**–**21**^a^

**product**	**starting compound**	**reagents and solvent**	**temperature and time**	**yield**
**1**	costunolide	*p*-toluenesulfonic acid, CH_2_Cl_2_	R.T., 4 h	52%
**2**	**1**	SeO_2_, *t*-BuOOH, CH_2_Cl_2_	R.T., 24 h	44%
**3**	**1**	(1) 4-methoxybenzyl alcohol, DBU	(1) R.T., 24 h	(1) 46%
		(2) DDQ, CH_2_Cl_2_	(2) R.T., 5 h	(2) 75%
**4**	costunolide	*p*-toluensulfonic acid, CH_2_Cl_2_	R.T., 4 h	34%
**5**	**4**	(1) 4-methoxybenzyl alcohol, DBU	(1) R.T., 24 h	(1) 42%
		(2) DDQ, CH_2_Cl_2_	(2) R.T., 6 h	(2) 86%
**6**	**4**	DDQ, CH_2_Cl_2_	reflux, 24 h	67%
**7**	**6**	(1) 4-methoxybenzyl alcohol, DBU	(1) R.T., 24 h	(1) 40%
		(2) DDQ, CH_2_Cl_2_	(2) R.T., 5 h	(2) 87%
**8**	**6**	SeO_2_, *t*-BuOOH, CH_2_Cl_2_	R.T., 24 h	44%
**9**	costunolide	*p*-toluensulfonic acid, CH_2_Cl_2_	R.T., 4 h	6%
**11**	DHC (**10**)	SeO_2_, *t*-BuOOH, CHCl_3_	R.T., 35 min	14%
**12**	DHC (**10**)	SeO_2_, *t*-BuOOH, CHCl_3_	R.T., 35 min	51%
**13**	DHC (**10**)	SeO_2_, *t*-BuOOH, CHCl_3_	R.T., 35 min	19%
**14**	DHC (**10**)	SeO_2_, *t*-BuOOH, CHCl_3_	R.T., 35 min	21%
**15**	**14**	SeO_2_, *t*-BuOOH, CHCl_3_	R.T., 24 h	28%
**16**	DHC (**10**)	(1) 4-methoxybenzyl alcohol, DBU	(1) R.T., 48 h	(1) 65%
		(2) DDQ, CH_2_Cl_2_, H_2_O	(2) 0 °C, 6 h	(2) 86%
**17**	DHC (**10**)	(1) CH_3_CHO, *h*ν	(1) R.T., 1 h	(1) 70%
		(2) NaBH_4_, MeOH	(2) 0 °C, 1 h	(2) 96%
**18**	11 + 12	PCC, CHCl_3_	0 °C, 1 h	52%
**19**	**4**	SeO_2_, *t*-BuOOH, CH_2_Cl_2_	R.T., 24 h	4%
**20**	**9**	SeO_2_, *t*-BuOOH, CH_2_Cl_2_	R.T., 24 h	5%
**21**	**9**	SeO_2_, *t*-BuOOH, CH_2_Cl_2_	R.T., 24 h	13%

a R.T.: room temperature.

Compounds **1**, **4**, and **9** are eudesmanolides produced by plants, and these were synthesized from costunolide (200 mg, 0.86 mmol) in the same acid–base reaction with *p*-toluenesulfonic acid (41 mg, 0.24 mmol) in CH_2_Cl_2_ (5 mL) (Figure 3).^19^ After stirring for 4 h, the reaction was quenched with saturated aqueous NaHCO_3_ (25 mL) and the products were extracted with CH_2_Cl_2_ (3 × 20 mL). The combined organic layers were washed with brine (65 mL), dried over anhydrous Na_2_SO_4_, and filtered. The crude product was purified by column chromatography with an *n*-hexane/ethyl acetate gradient (1:0–17:3 *v*/*v*) to give 104 mg of **1** (52% yield), 68 mg of **4** (34% yield), and 12 mg of **9** (6% yield). The structures were confirmed by comparing their ^1^H NMR spectra (Figures S7.1, S7.3, and S7.7) with those reported for β-, α-, and γ-cyclocostunolide, respectively.^19,35^

Compound **6** was obtained from the reaction of α-cyclocostunolide (**4**) with the oxidant 2,3-dichloro-5,6-dicyano-*p*-benzoquinone (DDQ) (Figure 3),^35^ and the structure was confirmed by comparing its ^1^H NMR spectrum (Figure S7.6) with that reported for 3-deoxybrachylaenolide.^36^

### 2.3 Synthesis of the Eudesmanolide Derivatives

**Figure 4:**
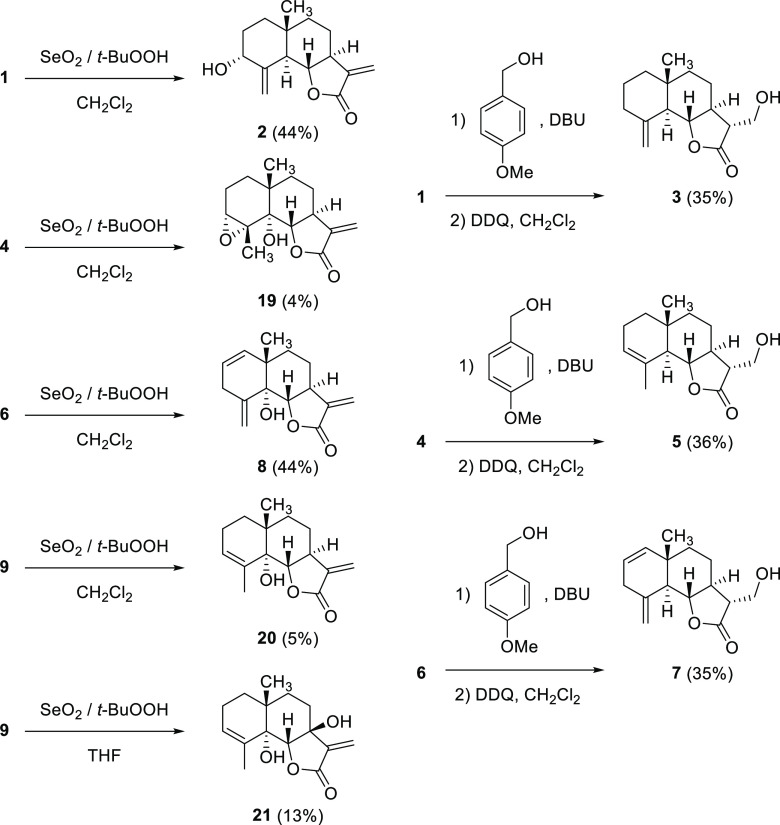
Reactions applied to eudesmanolides **1**, **4**, **6**, and **9** to obtain hydroxylated derivatives in the A-ring (left column) and at C-13 (right column).

Eudesmanolide derivatives hydroxylated in the A-ring (**2** and **8**) and at C-13 (**3**, **5**, and **7**) were synthesized following different strategies, namely, oxidation with SeO_2_ and *tert*-butyl hydroperoxide (*t*-BuOOH) or formation of an ether on the lactone by Michael addition and subsequent oxidation and hydrolysis (Figure 4), respectively.

#### 2.3.1 Synthesis of **2** and **8**

These compounds were prepared by hydroxylation of compounds **1** and **6**, respectively (Figure 4). To a solution of **1** (46.8 mg, 0.201 mmol) or **6** (16.4 mg, 0.071 mmol) in dry CH_2_Cl_2_ (0.5 mL) were added SeO_2_ (0.5 mmol) and *t*-BuOOH (70% *v*/*v* in H_2_O, 2 mmol) for each mmol of **1** or **6**. The mixture was stirred for 24 h and then filtered under vacuum through a filter funnel with a glass sinter containing silica gel. The filtrate was concentrated and purified by column chromatography (*n*-hexane/ethyl acetate gradient 1:0–7:3, *v*/*v*). Compound **2** (21.9 mg, 0.088 mmol) and compound **8** (7.63 mg, 0.031 mmol) were obtained in 44% yield. Compounds **2** and **8** are reported here for the first time and their spectroscopic data are provided as Supporting Information (see S1 and S2).

#### 2.3.2 Synthesis of **3**, **5**, and **7**

These compounds were obtained in two steps from compounds **1**, **4**, and **6**: formation of an ether at C-13 with 4-methoxybenzyl alcohol and 1,8-diazabicyclo[5.4.0]-undec-7-ene (DBU), and subsequent reaction with DDQ (Figure 4). Following the published procedure, compounds **3**, **5**, and **7** were obtained in 35–36% global yield and their structures were confirmed by comparison of their ^1^H NMR spectra (Figures S7.2, S7.4, and S7.6).^19,35^

### 2.4 Synthesis of Guaianolides

Hydroxylated derivatives in the A-ring (**11**–**15**), C-13 (**16**) and C-16 (**17**), and dehydrozaluzanin C (**18**) were synthesized from DHC (**10**) following different strategies.

**Figure 5:**
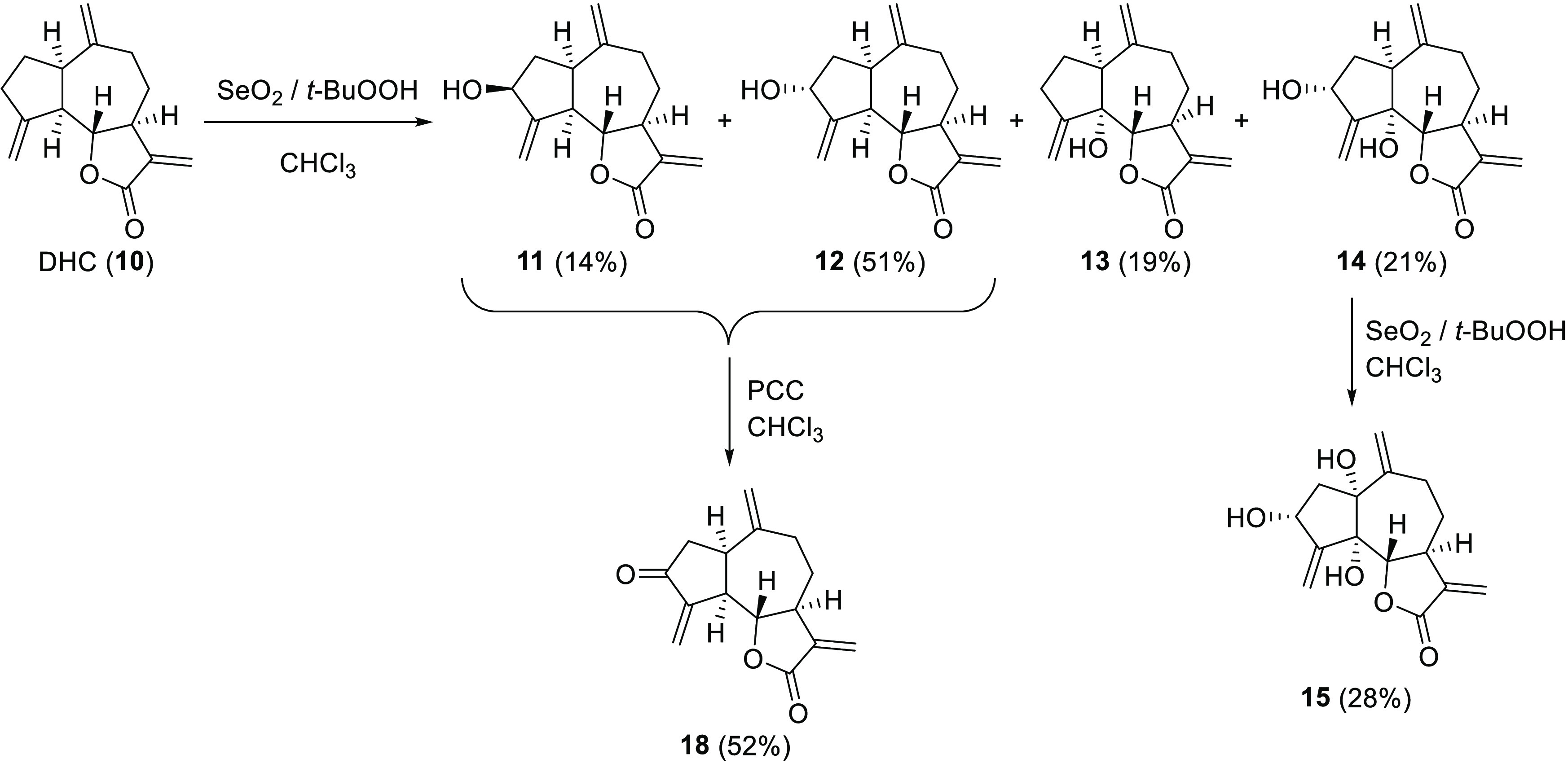
Synthesis of guaianolides **11**–**15** and dehydrozaluzanin C (**18**).

Compounds **11**–**14** were obtained by allylic oxidation of a solution of DHC (100 mg, 0.434 mmol) in CHCl_3_ (15 mL) with SeO_2_ (12.0 mg, 0.11 mmol) and subsequent addition of *t*-BuOOH (400 μL, 70% *v*/*v* in H_2_O, 2.8 mol) at a rate of 0.04 mL per minute (Figure 5).^37^ After 35 min, the mixture was filtered through silica gel and purified by column chromatography with an *n*-hexane/ethyl acetate gradient (9:1–6:4, *v*/*v*) to give zaluzanin C (**11**, 15.0 mg, 0.061 mmol, 14% yield), isozaluzanin C (**12**, 55.2 mg, 0.224 mmol, 51% yield), **13** (20.5 mg, 0.083 mmol, 19% yield), and **14** (24.5 mg, 0.093 mmol, 21% yield). The structures were confirmed by comparison of their ^1^H NMR spectra (Figure S8.2–8.5) with previously reported data.^37^

Derivative **15** was obtained by application of the same reaction to compound **14** (50 mg, 0.19 mmol) in CHCl_3_ (10 mL) and treatment with SeO_2_ (14.0 mg, 0.13 mmol) and *t*-BuOOH (70% *v*/*v* in H_2_O, 100 μL, 0.7 mol, added at a rate of 0.04 mL per minute). After a reaction time of 24 h, purification of the crude product by column chromatography with an *n*-hexane/ethyl acetate gradient (8:2–2:8, *v*/*v*) gave compound **15** (4.73 mg, 0.017 mmol, 28% yield). The structure was confirmed by comparison of its ^1^H NMR spectroscopic data (Figure S8.6) with the previously published data.^37^

**Figure 6:**
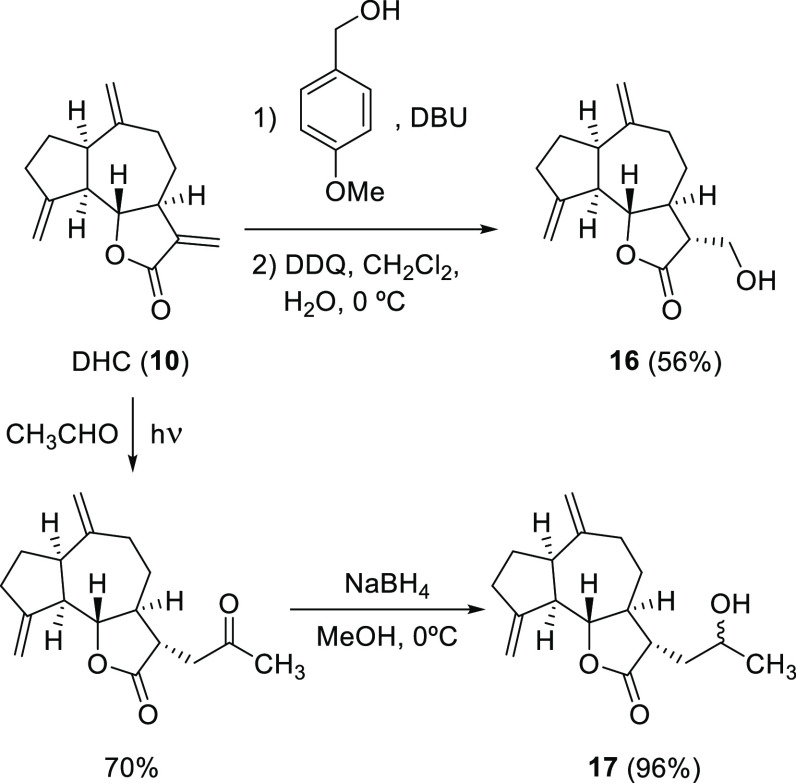
Synthesis of the hydroxylated derivatives of DHC at C-13 (**16**) and C-16 (**17**).

Compound **16** was synthesized following the procedure described in Section 2.3.2 for the synthesis of derivatives **3**, **5**, and **7** to give the product with a global yield of 56% (0.861 mmol) from 200 mg of DHC (Figure 6).^19^

Compound **17**, a derivative of DHC (**10**) with a 2-hydroxypropyl group at C-11, was obtained by photochemical addition of acetaldehyde at C-13 followed by the reduction of the carbonyl group with NaBH_4_ (Figure 6).^38^ Treatment of DHC (100 mg, 0.434 mmol) gave the desired methyl ketone in 70% yield. In the second step, the methyl ketone (20.0 mg, 0.073 mmol) was dissolved in anhydrous MeOH (5.0 mL) at 0 °C (water/ice bath) under N_2_ and NaBH_4_ (50.3 mg, 1.4 mmol) was added. The reaction mixture was stirred for 1 h. Distilled water (10 mL) was added, and the product was extracted with ethyl acetate (3 × 15 mL). The combined organic extracts were dried over anhydrous Na_2_SO_4_ and filtered. The crude product was purified by column chromatography with an *n*-hexane/ethyl acetate gradient (9:1–6:4, *v*/*v*) to give a 2:1 epimeric mixture of **17** in 96% yield (19.4 mg, 0.07 mmol), 67% global yield. An unsuccessful attempt was made to purify the epimeric mixture by analytical HPLC. The structure of **17** was confirmed by comparison of its ^1^H NMR spectroscopic data (Figure S8.8) with previously reported data.^38^

The synthesis of dehydrozaluzanin C (**18**) was performed following the procedure described in the literature^23^ (Figure 5). A mixture of **11** and **12** (100 mg, 0.406 mmol) was dissolved in CHCl_3_ (10 mL). The mixture was stirred and pyridinium chlorochromate (PCC) (180 mg, 0.834 mmol) was added at 0 °C (ice/water bath). The mixture was stirred for 1 h at room temperature. The mixture was filtered through silica gel, and the filtrate was purified by column chromatography with an *n*-hexane/ethyl acetate gradient (9:1–6:4, *v*/*v*) to give **18** in 52% yield (51.8 mg, 0.21 mmol). The structure was confirmed by comparison of the ^1^H spectroscopic data (Figure S8.9) with those of dehydrozaluzanin C.^23^

### 2.5 Bioassays

#### 2.5.1 Phytotoxicity on Etiolated Wheat Coleoptiles

Products were tested at concentrations of 1000, 300, 100, 30, and 10 μM on etiolated wheat coleoptiles (*Triticum aestivum* L. cv. Burgos, seeds provided by FITÓ S.A., Spain) following reported protocols.^25,39^ The commercial herbicide Logran Extra 60 WG was used as positive control in the same range of concentrations (1000–10 μM) in relation to the active compounds in the herbicide composition (59.4% terbutryn and 0.6% triasulfuron, *w*/*w*). Results are presented as percentage elongation against the negative control (buffered aqueous solution).

#### 2.5.2 Phytotoxicity against Weeds

Products were tested at concentrations of 1000, 300, 100, 30, and 10 μM on *A. viridis* L., *E. crus-galli* L., and *L. perenne* L. seeds following reported protocols.^25,40^ Seeds were provided by Herbiseed Co. (Twyford, England) and were preserved at 5 °C before use. The commercial herbicide Logran Extra 60 WG was used as positive control in the same range of concentrations (1000–10 μM) in relation to the active compounds in the herbicide composition (59.4% terbutryn and 0.6% triasulfuron, *w*/*w*). Statistical analysis was performed by Welch’s test, with significance levels established at 0.01 and 0.05.

#### 2.5.3 Calculation of IC_50_ and Clog *P* Values

IC_50_ values were calculated by fitting the activity data to a sigmoidal dose–response model using GraphPad Prism 5.00 software.^25^

Lipophilicity is a useful parameter in bioactivity studies of structurally related compounds as it can highlight trends to begin to specify modes of action, including phytotoxicity studies. For the discussion, lipophilicity is expressed by the Clog *P* calculation method as implemented in ChemBioDraw Ultra 21.0 software.^19,41^

## 3 Results and Discussion

### 3.1 Synthesis of the New Derivatives

Eudesmanolides **2** and **8** were synthesized for the first time by a reaction that has also been applied to other eudesmanolides (spectroscopic data provided as the Supporting Information), but the low yields prevented the study of the products (**19**–**21**, Figure 4) in bioassays. These derivatives are hydroxylated in the A-ring, and this substitution pattern was achieved due to the presence of a double bond in this ring. The hydroxylation strategy involved oxidation of the double bonds with SeO_2_ and *t*-BuOOH.^42,43^ This allowed the introduction of hydroxyl groups in some of the positions closest to the double bond, which in some cases generated bond isomerization.

The reaction of **1** gave compound **2** (monohydroxylated product at C-3), and reaction of **6** gave compound **8** (monohydroxylated product at C-5). These two compounds are formed in a three-step mechanism that involves a pericyclic reaction, namely, an Alder-ene reaction, a [2,3] sigmatropic rearrangement, and final hydrolysis. In a previous study, this reaction was applied to a eudesmanolide that is structurally related to **1**, obtaining the corresponding homologue of compound **2**.^44^ The synthesis of the C-3 epimer of **2** was previously published, and this approach employed the sesquiterpene lactone hanphyllin as starting material.^45,46^ The differences found in the ^1^H NMR spectrum of compound **2** and the opposite [α]_D_ value obtained in comparison to the data reported for its C-3 epimer confirm that compound **2** was the product (see S1). The stereochemistry of C-3 was also deduced from the coupling constant values (*J*) between H-3 and both H-2 (2.3 Hz). If one assumes that the A-ring has a chair conformation, these *J* values indicate an equatorial position for H-3 since an axial position would give an estimated *J* value of 8–10 Hz with one of the H-2 protons.

Compound **8** has not previously been reported in the literature. Its structure was mainly assigned by comparison of its ^1^H NMR spectrum (see S2) with that of **6**. Hydroxylation of C-5 was deduced by the absence of the H-5 signal (at δ 2.50 in compound **6**) and the simpler multiplicity of the H-6 signal (from *dd* at δ 3.98 to *d* at δ 4.24). The α-orientation of the hydroxyl group was deduced from the shift of H-14 (δ 0.93 ppm for compound **8**), which is very similar to that of compound **6** (δ 0.85 ppm). If the hydroxyl group had a β-orientation, its proximity to the angular methyl group (C-14) would have led to a more marked change in chemical shift. Indeed, such a change was observed for the eudesmanolides santamarine, gallicadiol, and 5-epigallicadiol, for which the β-orientation provoked a change in shift in the H-14 signal of around 0.50 ppm, whereas very close values (0.04 ppm of difference) at around δ 0.90 ppm were observed for the α-orientated and the nonhydroxylated compounds.^25,47^

This oxidation strategy, which provided hydroxylated eudesmanolide derivatives **1** and **6** in good yield, was applied to eudesmanolides **4** and **9**. As a result, several new hydroxylated eudesmanolides (**19**–**21**) were obtained, albeit in low yield, and these were characterized (NMR data and spectra are provided in S3–S5).

The reaction of eudesmanolide **4** gave compound **19**, which contains an epoxide ring in addition to the new hydroxyl group at C-5 (Figure 4). The structure of **19** was deduced by comparison of its ^1^H NMR spectrum (Figure S3.1) with that of starting material **4**. The *d* multiplicity of H-6, instead of *dd*, the change in chemical shift from δ 3.66 to δ 4.09, and the presence of a signal for H-7 all indicated the hydroxylation of C-5. The hydroxyl group must have an α-orientation on the basis of the same spectroscopic data as outlined above for the elucidation of compound **8**, as significant changes were not observed (a change of only 0.07 ppm) between the H-14 signals. The epoxide between C-3 and C-4 was deduced by the new shift of the H-3 signal (from δ 5.36 for **4** to 3.19 for **19**) and the C-4 signals in the ^13^C NMR spectra (from δ 122.4 to 61.2). The α-orientation of the epoxide ring was determined by the NOE effects (Figure S3.3) observed between the H-15 signal (δ 1.56) with H-6 and H-14 (both β-oriented), which indicate that the methyl group C-15 also has a β orientation. An NOE effect was also observed between H-15 and H-3 signals, and this shows that H-3 has a β orientation.

The reaction of compound **9** gave the monohydroxylated product **20** (Figure 4) in low yield. It is envisaged that this reaction occurs by a slightly different mechanism than that hypothesized for compounds **2** or **8**, with the second step involving a [1,2] migration that would explain the different position of the double bond. Compound **20** is reported here for the first time. The C-1-hydroxylated derivative of **20** was synthesized in another study using the same reagents.^48^ The structure of compound **20** was deduced from its ^1^H NMR spectrum (see S4), which showed a new signal at δ 5.51 that was not present in the spectrum of the starting material **9**, thus denoting the change in the position of the double bond to C_3_=C_4_. The position at C-5 and the α-orientation of the hydroxyl group were elucidated by analogy to the observations detailed for the structural characterization of compound **8**.

In an attempt to improve the yield of compound **20**, the reaction was performed in dry tetrahydrofuran (THF) instead of CH_2_Cl_2_ and the double-hydroxylated product **21** was obtained in low yield. This product is similar to **20**, but it has an additional β-hydroxyl group at C-7 and it has not been reported previously. Hydroxylated eudesmanolides at C-7 are not commonly isolated, and previous examples include 7α-hydroxyfrullanolide and subspicatolide.^49,50^ Nevertheless, to the best of our knowledge, examples of 7β-hydroxylated eudesmanolides have not been reported as natural products. A strategy for the synthesis of these particular eudesmanolides was developed,^51,52^ although it differs significantly from the approach presented here. The addition of a hydroxyl group at C-7 in compound **21** was ascertained by the absence of the H-7 signal (δ 2.58 in compound **9** or δ 3.34 in compound **20**), the *s* multiplicity of the H-6 signal, and the marked change in chemical shift of the H-6 and H-13 signals (see S5). The β-orientation of the hydroxyl group at C-7 was inferred from the shift for H-6 (δ 5.15). This value was δ 4.08 for compound **20** (same structure but without a hydroxyl group at C-7), with a significant difference observed between the two values. This difference can be explained by a similar orientation of H-6 (with a characteristic β orientation) and H-7 in compound **21**. The spectroscopic data published by Gonzalez Collado et al. for C-7 hydroxylated eudesmanolides showed similar changes in the H-6 shifts,^36^ and in another reference, the H-6 shift for compounds with an α orientation was similar to that of the same signal for the nonhydroxylated molecule at C-7.^53^ This information corroborates the structure assigned to **21**.

### 3.2 Phytotoxicity on Etiolated Wheat Coleoptiles

**Figure 7:**
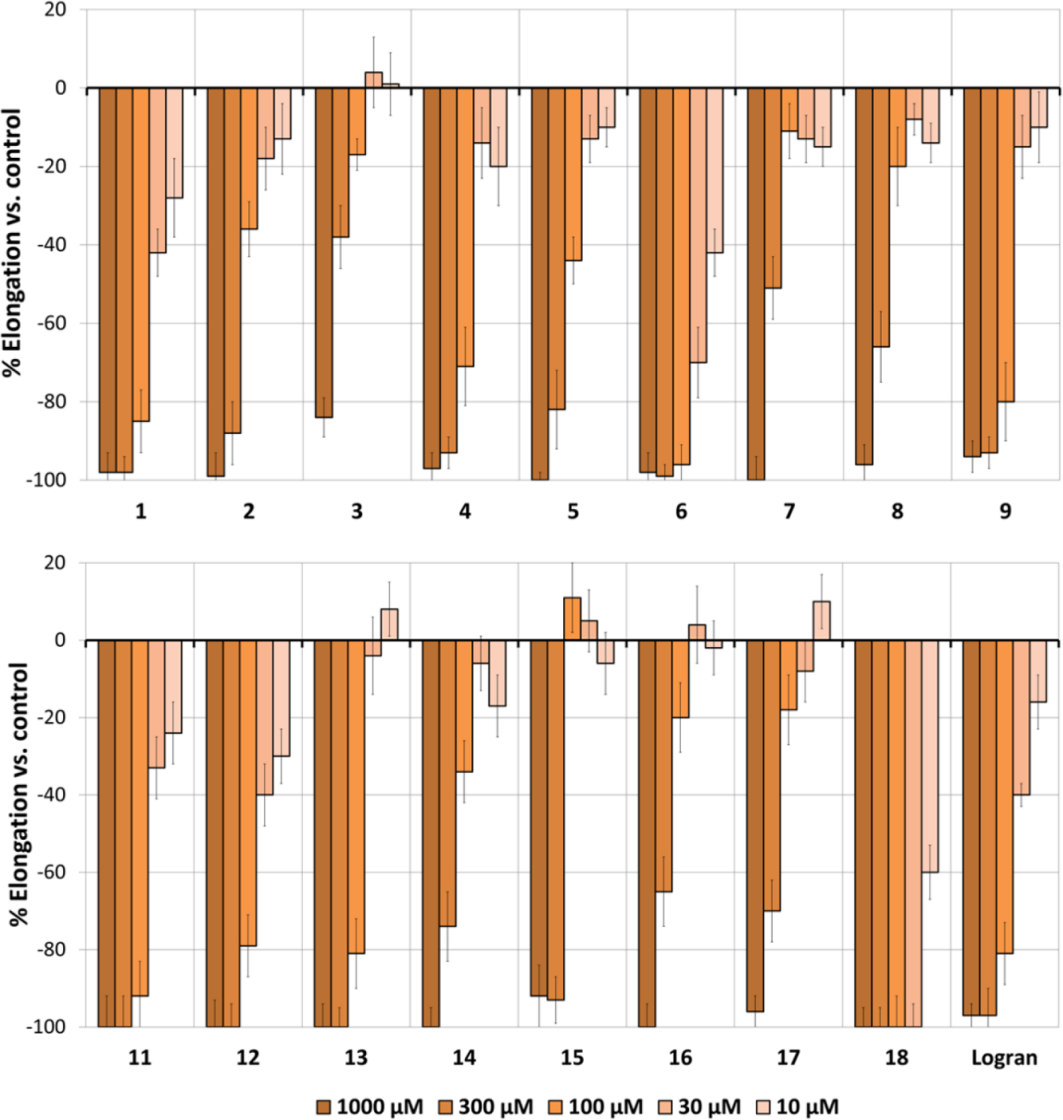
Phytotoxicity profiles obtained for eudesmanolides **1**–**9**, guaianolides **11**–**18**, and the positive control (Logran) in the etiolated wheat coleoptile bioassay. Positive values indicate stimulation of growth vs the negative control and negative values indicate inhibition. Error bars represent the standard error of the mean.

The phytotoxicity of eudesmanolides **1**–**9** and guaianolides **11**–**18** were evaluated in the wheat coleoptile bioassay (Figure 7), which allows a rapid and reliable study of the inhibition caused on the growth of etiolated coleoptiles. The commercial herbicide Logran was used as positive control.

All of the compounds generated high inhibition at 1000 μM with most values over 90%. At 300 μM, these levels were retained by a significant number of compounds, with the most active examples (**1**, **4**, **6**, **9**, **11**–**13**, and **18**) showing high inhibition percentages even at 100 μM. Compound **6** and especially **18** were the only ones that displayed significant inhibition at the lowest concentrations (42 and 60%, respectively), with IC_50_ values of 13.2 and 5.33 μM, respectively.

On comparing the results for eudesmanolides **1**–**9**, the most active compounds were those that did not contain a hydroxyl group (**1**, **4**, **6**, and **9**). The IC_50_ values (Table 2) in these cases were close to or even better than that obtained for the herbicide Logran (39.1 μM) and, in the case of **6**, significantly better (13.2 μM) due to the maintenance of high inhibition at the lowest concentrations. In general, the most active compounds also have the highest lipophilicity (Clog *P*) values (Table 2). Thus, the presence of the hydroxyl group decreases the Clog *P* values by around 1 unit, and this could explain the different behavior as being due to a lower solubility in cell membranes. Clear SAR involving the position of the hydroxyl group could not be defined.

**Table 2:** IC_50_ Values of Compounds **1**–**18** in the Phytotoxicity Bioassay on Coleoptiles and Lipophilicity Values Calculated as Log *P* and Clog *P*^a^

**compound**	**IC**_**50**_**(μM)**	**Clog** *P*
**1**	31.7	3.27
**2**	124.6	1.38
**3**	376.3	2.07
**4**	66.1	3.27
**5**	118.8	2.07
**6**	13.17	2.79
**7**	320.7	1.59
**8**	229.6	1.68
**9**	56.1	3.47
DHC (**10**)^19^	28.8	2.79
**11**	36.0	0.90
**12**	36.1	0.90
**13**	62.9	1.68
**14**	169.0	0.45
**15**	222.1	0.07
**16**	217.7	1.59
**17**	193.0	2.10
**18**	5.33	0.96
Logran	39.1	

a *R*^2^ values were in the range 0.95–0.99.

Guaianolides **11**–**18** generally showed better inhibitory activity than the hydroxylated eudesmanolides. Compound **18**, which contains a characteristic unsaturated ketone in the A-ring, showed the highest inhibition, with a value of 100% even at the fourth concentration (30 μM) and 60% at the minimum concentration evaluated. Its IC_50_ value was the lowest found in this study. Among the hydroxylated derivatives, the best results were obtained for compounds monohydroxylated at C-3 (**11** and **12**, IC_50_ = 36 μM), followed by the compound hydroxylated at C-5 (**13**, IC_50_ = 62.9 μM).

The activity of DHC (**10**), the nonhydroxylated parent of guaianolides **11**–**18**, was published previously (IC_50_ = 28.8 μM).^19^ The introduction of a carbonyl group in the A-ring led to a significant improvement in activity. It is believed that the presence of an α,β-unsaturated system is strongly linked to the bioactivity of sesquiterpene lactones, and further studies regarding the mechanism of action would be necessary to find the reason for its higher activity. The authors consider that it is possible that the presence of a second unsaturated system is responsible for the increased activity of compound **18**, but it is also feasible that the activity of **18** is related to a different mechanism of action owing to the presence of these two unsaturated systems. The introduction of the hydroxyl groups in the A-ring did not lead to a significant decrease in the activity level when compared to DHC. On considering the results for the monohydroxylated guaianolides in the A-ring (**11**–**13**), functionalization at C-5 seems to have a detrimental effect whereas the orientation of the hydroxyl group at C-3 did not cause significant changes in activity. When two (**14**) or three (**15**) hydroxyl groups are introduced, the inhibition levels decreased to a greater extent. The latter results can be explained in terms of physicochemical properties on examining the calculated Clog *P* values (Table 2), since the most active compounds have values around 0.90, which is higher than those of the less active compounds (0.45 for **14**, and 0.04 for **15**). Thus, monohydroxylated derivatives would pass through the cell membranes more efficiently to the site of action. In the cases of compounds **16** and **17**, the removal of the γ-butyrolactone system led to decreased activity when compared to DHC.

### 3.3 Phytotoxicity against Weeds

**Figure 8:**
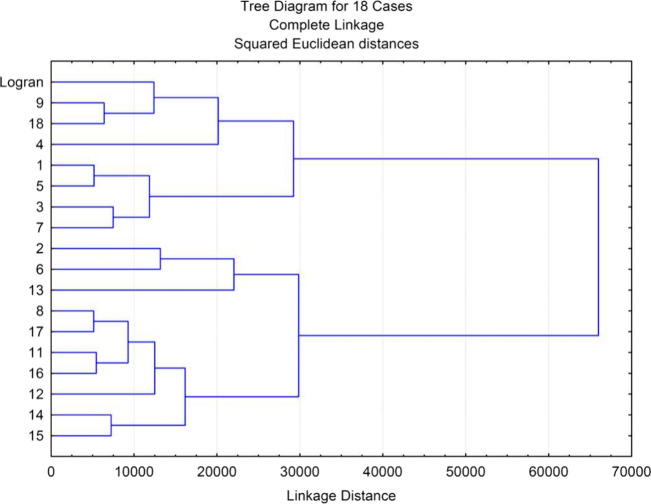
Cluster analysis of the phytotoxicity of compounds **1**–**9**, **11**–**18**, and the herbicide Logran (positive control) against *Amaranthus viridis*, *Echinochloa crus-galli*, and *Lolium perenne* growth.

The phytotoxicity of the compounds was evaluated against three specific weed species: *Amaranthus viridis* L., *Echinochloa crus-galli* L., and *Lolium perenne* L. The commercial herbicide Logran was used as positive control. In an effort to facilitate comparison of the results, IC_50_ values were calculated for shoot and root lengths (Table 3) and a cluster analysis was performed (Figure 8). Both eudesmanolides and guaianolides were active on the tested species. Germination rates consistently ranged between 80 and 90%. γ-Cyclocostunolide (**9**) was among the most active eudesmanolides in all cases, and dehydrozaluzanin C (**18**) was the most active guaianolide.

**Table 3:** IC_50_ Values (μM) of Compounds **1**–**18** in the Phytotoxicity Bioassay against *Amaranthus viridis*, *Echinochloa crus-galli*, and *Lolium perenne* Weeds^a^

	Amaranthus viridis		Echinochloa crus-galli		Lolium perenne	
**compound**	**IC**_**50**_ **(root)**	**IC**_**50**_**(shoot)**	**IC**_**50**_**(root)**	**IC**_**50**_**(shoot)**	**IC**_**50**_ **(root)**	**IC**_**50**_**(shoot)**
**1**	153.8	120.1	>1000	>1000	>1000	869.2
**2**	407.3	540.4	198.9	202.6	995.3	>1000
**3**	34.7	33.7	783.9	>1000	>1000	>1000
**4**	5.2	5.8	486.6	285.6	>1000	869.9
**5**	125.7	152.0	953.3	611.5	>1000	>1000
**6**	208.2	163.3	33.9	125.0	>1000	>1000
**7**	61.2	42.0	>1000	450.4	>1000	>1000
**8**	369.0	487.2	>1000	872.9	>1000	>1000
**9**	31.6	37.9	173.8	152.2	705.6	514.1
DHC (**10**)^26,27^	75.7	93.9	843.8	604.6	609.6	196.8
**11**	252.2	315.8	510.5	659.9	>1000	>1000
**12**	211.6	288.2	>1000	629.8	>1000	>1000
**13**	191.9	189.6	223.0	288.3	502.2	>1000
**14**	383.1	826.3	643.8	291.9	>1000	>1000
**15**	819.0	>1000	903.6	720.8	>1000	>1000
**16**	204.9	347.2	414.1	>1000	924.7	>1000
**17**	323.4	412.8	>1000	>1000	>1000	>1000
**18**	37.2	49.4	298.6	448.3	593.0	>1000
Logran	27.8	85.7	167.5	288.2	99.1	571.4

a *R^n^* values were in the range 0.95–0.99.

Germination was not significantly affected, whereas phytotoxic effects were observed on root and shoot lengths. A general view of the IC_50_ values in Table 3 shows that the best results were obtained for the inhibition of *A. viridis* and *E. crus-galli*. In general, eudesmanolides were more active than guaianolides, especially at lower concentrations, with the exception of guaianolide **18** and in some cases **13**. Cluster analysis and IC_50_ values identified eudesmanolide **9** as the most active compound, followed by **18** and **4**. Therefore, eudesmanolides without hydroxyl groups and with an endocyclic double bond in the A-ring (**4** and **9**) are among the most phytotoxic compounds, while eudesmanolides with an exocyclic double bond (**1** and **6**) and a hydroxyl group (**2**, **3**, **5**, **7**, and **8**) were less active. However, singly hydroxylated eudesmanolides could provide improved activity depending on the weed species, as observed for **2** and **7**.

#### 3.3.1 Phytotoxicity against *Amaranthus viridis*

**Figure 9:**
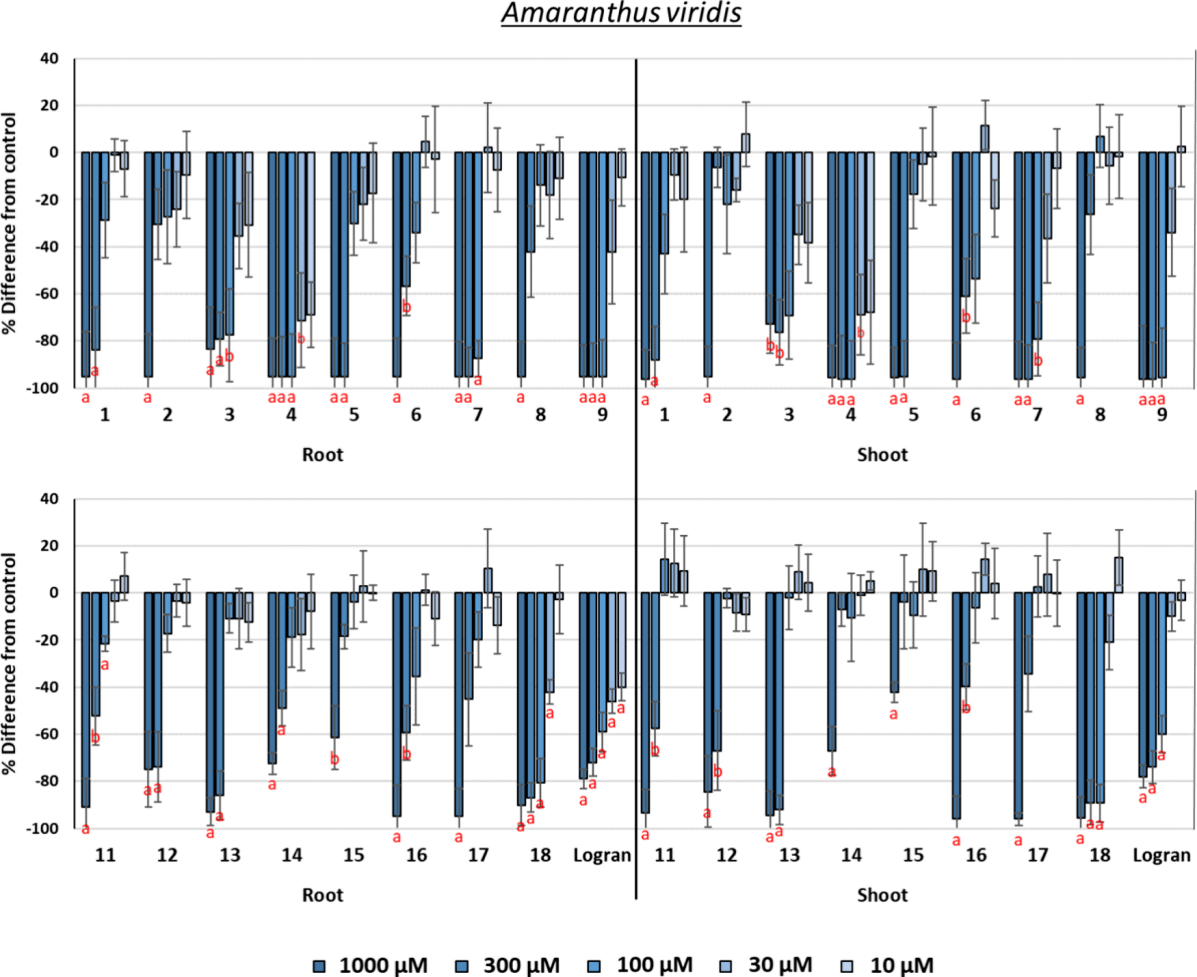
Phytotoxicity of eudesmanolides **1**–**9**, guaianolides **11**–**18**, and the herbicide Logran (positive control) against the root and shoot growth of *Amaranthus viridis*. Positive values indicate stimulation of growth vs the negative control, and negative values indicate inhibition. Significance levels: *p* < 0.01 (a), or 0.01 < *p* < 0.05 (b). Error bars represent the standard error of the mean.

The results are graphically depicted in Figure 9. Eudesmanolides generally showed better phytotoxicity than guaianolides, particularly at the lowest concentrations. All of the eudesmanolides apart from **3**, as well as some of the guaianolides, achieved inhibitions close to 100% at different concentrations. The best activity on both root and shoot growth, with IC_50_ values of 5.2 and 5.8 μM, respectively, was achieved by α-cyclocostunolide (**4**). These IC_50_ values are markedly lower than those of the herbicide Logran (27.8 and 85.7 μM). Eudesmanolides **3** and **9**, along with the guaianolide dehydrozaluzanin C (**18**), were also very active, with IC_50_ values in the range 31.6–49.4 μM. Thus, natural products such as **4**, **9**, and **18** would be of great interest for the development of herbicides against *A. viridis*.

It was concluded that hydroxylation of the A-ring decreases the phytotoxicity of eudesmanolides and guaianolides. A higher number of hydroxyl groups in guaianolides led to a decrease in activity (**10** > **11**–**13** > **14** > **15**). This result is related to a decrease in the lipophilicity to values closer to zero (Table 2). The orientation of the hydroxyl group in compounds **11** and **12** proved to have little influence on the activity. The hydroxylation of C-13 improved the phytotoxicity of eudesmanolides with an exocyclic double bond (**3** > **1** and **7** > **6**), with opposite behavior in cases where the double bond is endocyclic (**4** > **5**). This latter behavior was also observed for the guaianolides hydroxylated at C-13 or C-16 (**10** > **16** and **17**). The presence of a conjugated carbonyl in the A-ring also improved the activity of the guaianolide structure (**18** > **10**).

#### 3.3.2 Phytotoxicity against *Echinochloa crus-galli*

**Figure 10:**
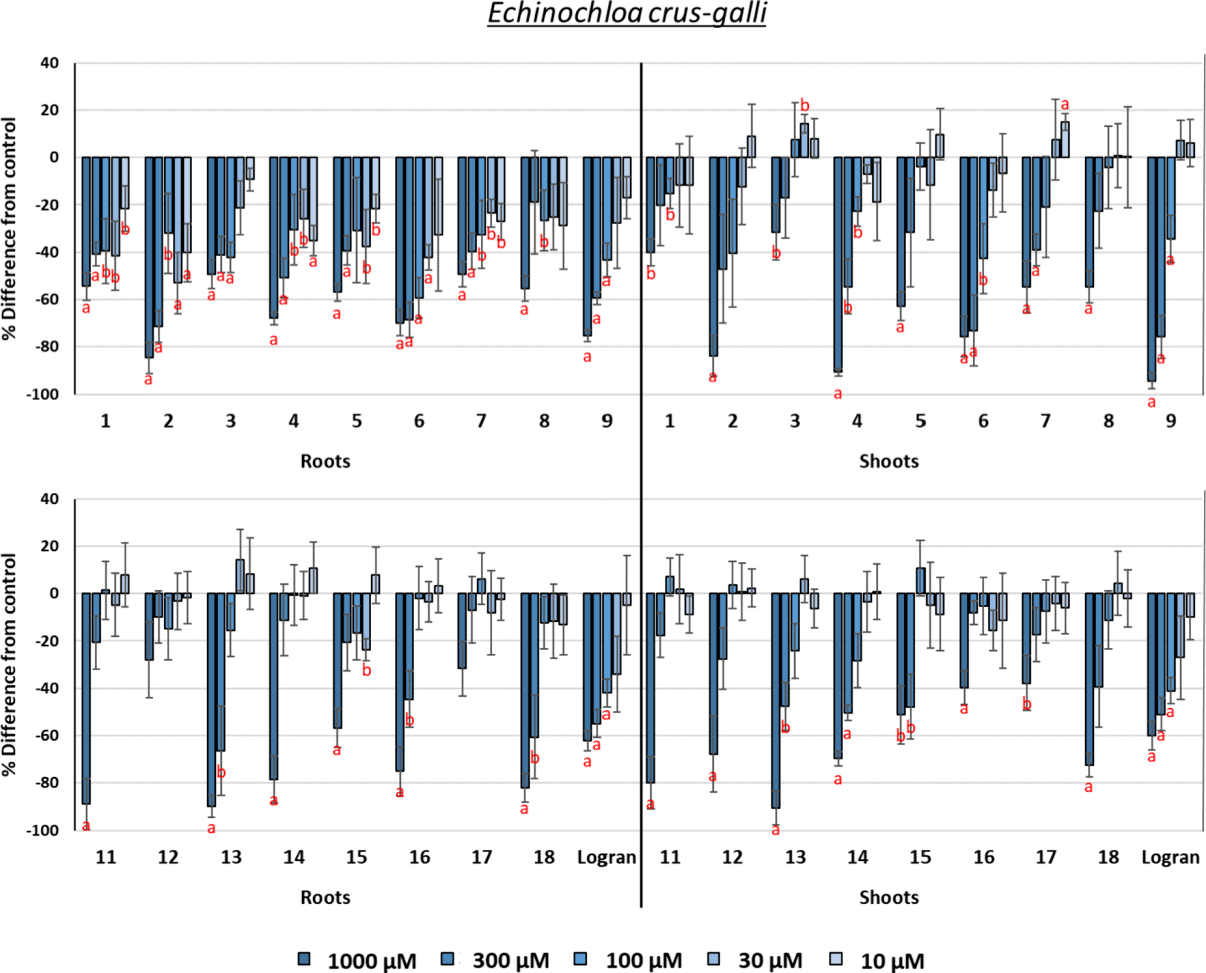
Phytotoxicity of eudesmanolides **1**–**9**, guaianolides **11**–**18**, and the herbicide Logran (positive control) against the root and shoot growth of *Echinochloa crus-galli*. Positive values indicate stimulation of growth vs the negative control, and negative values indicate inhibition. Significance levels: *p* < 0.01 (a), or 0.01 < *p* < 0.05 (b). Error bars represent the standard error of the mean.

The results are graphically depicted in Figure 10. Eudesmanolides generally showed better phytotoxicity than guaianolides, especially at the lowest concentrations. Root length was inhibited even at the lowest concentrations, although the shoot length was only inhibited at 1000 or 300 μM. Compound **6** was the most active example, with IC_50_ values (33.9 and 125.0 μM for root and shoot growth, respectively) that were significantly higher than those of the second most active compound (173.8 and 152.2 μM, compound **9**). However, other compounds showed a higher inhibition at the highest concentration when compared to **6** but with a more dramatic drop in activity with dilution, thus leading to poorer inhibition at low concentrations. In addition to **6** and **9**, compounds **2**, **13**, and **18** were very active against *E. crus-galli*. The good activity observed for compound **13**, whose only hydroxyl group is in an internal ring-closing position, is worth highlighting, and its comparison with the other hydroxylated guaianolides suggests that its better activity is related to an indirect involvement of the position of this hydroxyl group (e.g., better solubility or a decrease in steric or electronic hindrance).

Hydroxylation of the A-ring in eudesmanolides had different effects on comparing the pairs of compounds **1**/**2** and **6**/**8**. Given the lower activity of **1** and the notably better profile of **6**, it can be concluded that there is a positive influence of the additional double bond in this ring when the eudesmanolide is not hydroxylated. The hydroxylation of the A-ring in guaianolides (**11**–**15** vs **10**) generated a moderate improvement in the activity, with the exception of compound **12**. The change in the orientation of the hydroxyl group in this compound led to a marked improvement in the activity on root growth (**11** ≫ **12**), although the shoot growth was only slightly sensitive to this change. The activity on root growth, as also observed for *A. viridis*, also decreased when the number of hydroxyl groups increased, with the trihydroxylated guaianolide **15** having the lowest activity. Regarding hydroxylation of C-13, clear SAR conclusions could not be drawn due to the different trends observed depending on the structures of the compounds. The presence of an additional conjugated carbonyl in the A-ring improved the activity of the guaianolide structure (**18** > **10**).

#### 3.3.3 Phytotoxicity against *Lolium perenne*

**Figure 11:**
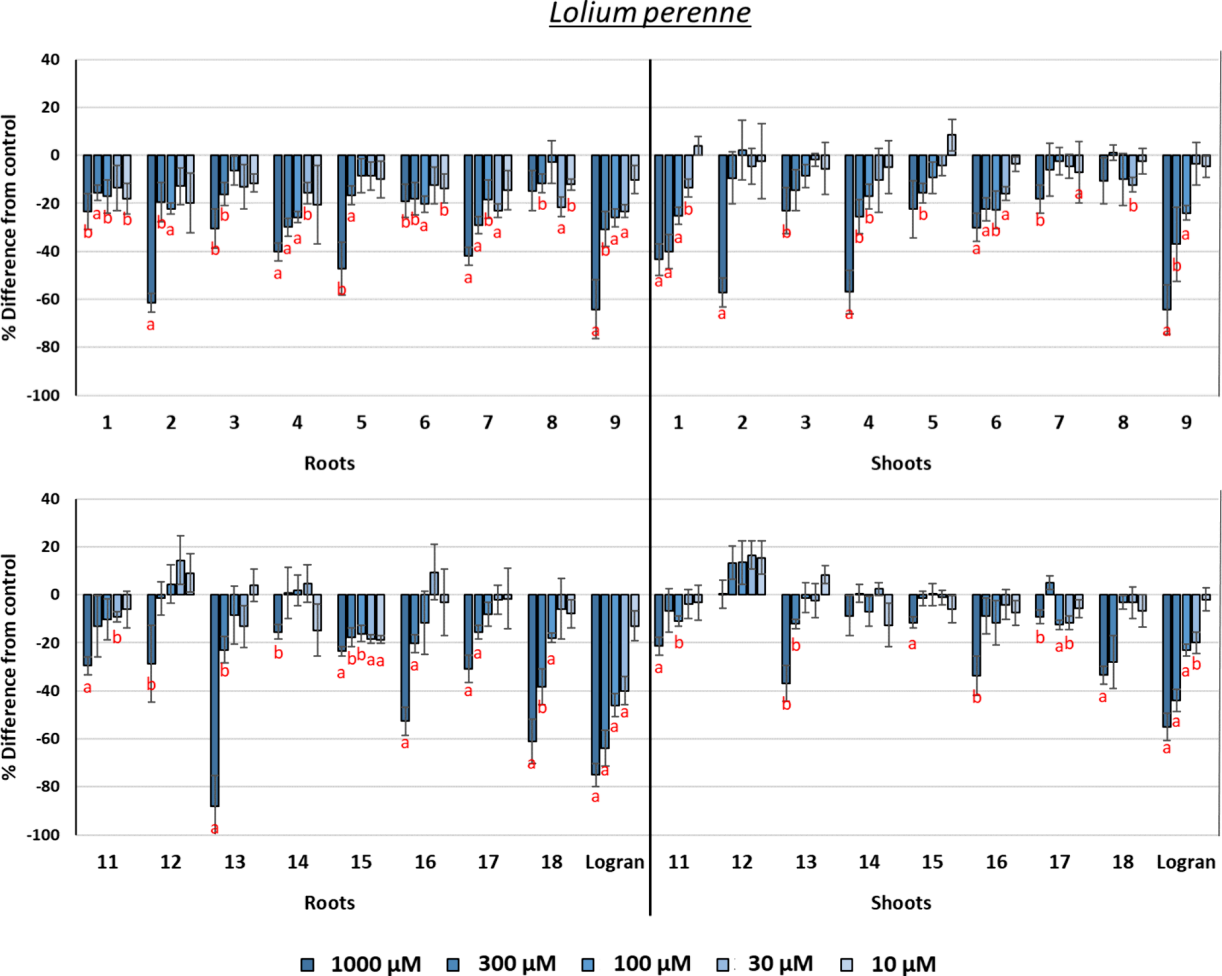
Phytotoxicity of eudesmanolides **1**–**9**, guaianolides **11**–**18**, and the herbicide Logran (positive control) against the root and shoot growth of *Lolium perenne*. Positive values indicate stimulation of growth vs the negative control, and negative values indicate inhibition. Significance levels: *p* < 0.01 (a), or 0.01 < *p* < 0.05 (b). Error bars represent the standard error of the mean.

The results are graphically depicted in Figure 11. The inhibition levels were generally low, but the inhibition achieved by some eudesmanolides and guaianolides at the highest concentration tested (1000 μM) are worth highlighting. Guaianolide **13** was the most active compound, and it showed the highest root growth inhibition value (88%), which is significantly higher than that of the next most active compounds (62–64% for **2** and **9**). Nevertheless, compounds **2** and **9**, unlike **13**, showed improved inhibition of shoot growth (57–64%). Compound **4** showed a similar effect. In terms of IC_50_ values, the best compounds against *L. perenne* were **13** (root growth) and **9** (root and shoot growth).

Hydroxylation of the A-ring did not generate significant changes in the activity of eudesmanolides, one exception being the improvement seen against root growth in the case of compound **2** vs **1**. In guaianolides—and taking into account the activity reported for DHC against *L. perenne* (around 60% inhibition on root and shoot growth at 1000 μM)^26^—this hydroxylation led to decreased activity, with the exception of the highly active compound **13**. The position of the hydroxyl group is relevant, and compound **13** was the most suitable example, as also found and discussed for *E. crus-galli*. Hydroxylation at C-13 led to decreased phytotoxicity on shoot growth both for eudesmanolides and guaianolides, and the unsaturated carbonyl group (**18**) retains the activity on root growth but decreases it to some extent on shoot growth.

**Figure 12:**
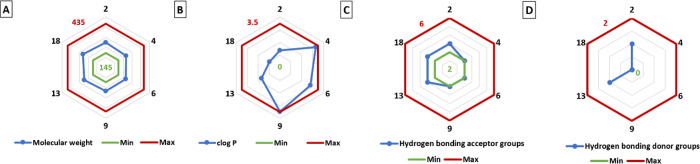
Graphs providing molecular descriptors for the most active compounds: (A) molecular weight; (B) Clog *P*, partition coefficient; (C) number of hydrogen bond acceptors; and (D) number of hydrogen bond donors. Red and green lines respectively indicate the minimum (min) and maximum (max) values found for commercially available herbicides.^54,55^

In summary, eudesmanolides and guaianolides provide suitable bioactive structures against three relevant weed species in agriculture (*A. viridis*, *E. crus-galli*, and *L. perenne*). A different degree of sensitivity has been observed between the species tested. Eudesmanolides generally showed improved phytotoxicity, especially at the lowest concentrations. Cluster analysis and IC_50_ values identified γ-cyclocostunolide (**9**) as the most active compound, followed by dehydrozaluzanin C (**18**) and α-cyclocostunolide (**4**), all of which are natural products. Thus, eudesmanolides without hydroxyl groups and with an endocyclic double bond in the A-ring, as well as a guaianolide with an unsaturated carbonyl group, are more suitable structures for use as lead compounds for the further development of natural product-based molecules with phytotoxic potential against the tested weeds. SAR have been discussed individually for each weed species. It was found that hydroxylation of the A-ring and C-13, as well as the position, number, and orientation of the hydroxyl groups, and the presence of an unsaturated carbonyl group can significantly influence the level of phytotoxicity on root and shoot growth. For eudesmanolides, compounds **4** and **9** were the most active on *A. viridis*, **6** and **9** on *E. crus-galli*, and **2** and **9** on *L. perenne*. In the case of guaianolides, compounds **13** and **18** were the most active on almost all of the weeds and parameters evaluated. The efficiency of compounds as agrochemicals is also contingent on diverse physicochemical properties associated with mobility, stability, and bioavailability. Consequently, the following parameters have been calculated and graphically depicted (Figure 12) for the most active compounds (**2**, **4**, **6**, **9**, **13**, and **18**), with the aim of illustrating how these properties are between the minimum and maximum values reported for commercially available herbicides, indicated within brackets as follows: molecular weight (145–435), partition coefficient (≤3.5), number of hydrogen bonding acceptor groups (2–6), number of hydrogen bonding donor groups (≤2), number of rotatable bonds (≤9), and number of bonds in aromatic rings (≤17).^54,55^ Notably, the number of rotatable bonds and bonds in aromatic rings are null in all the cases (data not shown in Figure 12).

In conclusion, the most active eudesmanolides and guaianolides can be considered as promising candidates for the development of herbicides based on natural products with the aim of achieving more efficient control of weed pests. Nevertheless, based on the presented results, it is necessary to avoid conclusively designating them as definitive model systems for herbicide development. Further studies regarding their applicability must be carried out, and this would include achieving high-yield production, ecotoxicological evaluation, and studies on a larger scale.^56,57^

## Supporting Information Available

Spectroscopic data and NMR spectra of the new products (S1–S5) and ^1^H NMR spectra of the previously reported compounds (S6–S8) (PDF)
