# Supplementary material for: Bioprospection of Phytotoxic Plant-Derived Eudesmanolides and Guaianolides for the Control of Amaranthus viridis, Echinochloa crus-galli, and Lolium perenne Weeds
Source: J Agric Food Chem. 2024 Jan 11;72(3):1797–810. doi: 10.1021/acs.jafc.3c06901 (PMC10811690; doi:10.1021/acs.jafc.3c06901)
Supplement: Supplementary file 1 — jf3c06901_si_001.pdf [file jf3c06901_si_001.pdf]

Bioprospection of phytotoxic plant-derived eudesmanolides and guaianolides for the control of *Amaranthus viridis*, *Echinochloa crus-galli* and *Lolium perenne* weeds

Jesús G. Zorrilla[1,2,*], David M. Cárdenas[2], Carlos Rial[2], José M.G. Molinillo[2], Rosa M. Varela[2,*], Marco Masi[1] and Francisco A. Macías[2]

[1] Department of Chemical Sciences, University of Naples Federico II, Complesso Universitario Monte S. Angelo, Via Cinthia 4, 80126 Naples, Italy

[2] Allelopathy Group, Department of Organic Chemistry, Facultad de Ciencias, Institute of Biomolecules (INBIO), University of Cadiz, C/Avenida República Saharaui, s/n, 11510 Puerto Real, Spain

* Corresponding authors. E-mail: jesus.zorrilla@uca.es ; rosa.varela@uca.es

Spectroscopic data and NMR spectra ($CDCl_3$, 500/125 MHz) of the new products

S1. Spectroscopic data of 3α-hydroxy-β-cyclocostunolide (**2**) 2

Figure S1.1. ^{1}H NMR spectrum 2

Figure S1.2. ^{13}C NMR spectrum 2

S2. Spectroscopic data of 5α-hydroxy-3-deoxybrachylaenolide (**8**) 3

Figure S2.1. ^{1}H NMR spectrum 3

Figure S2.2. ^{13}C NMR spectrum 3

S3. Spectroscopic data of 3α,4α-epoxy-5α-hydroxycyclocostunolide (**19**) 4

Figure S3.1. ^{1}H NMR spectrum 4

Figure S3.2. ^{13}C NMR spectrum 5

Figure S3.3. NOE 1D spectrum of the H-15 signal 5

S4. Spectroscopic data of 5α-hydroxy-α-cyclocostunolide (**20**) 6

Figure S4.1. ^{1}H NMR spectrum 6

Figure S4.2. ^{13}C NMR spectrum 6

S5. Spectroscopic data of 5α,7β-dihydroxy-α-cyclocostunolide (**21**) 7

Figure S5.1. ^{1}H NMR spectrum 7

Figure S4.2. ^{13}C NMR spectrum 7

^{1}H NMR spectra ($CDCl_3$, 500 MHz) of the previously reported compounds

S6. ^{1}H NMR spectrum of costunolide 8

S7. ^{1}H NMR spectra of eudesmanolides (**1**, **3-7** and **9**) 9

S8. ^{1}H NMR spectra of guaianolides (**10-18**) 12

S1. Spectroscopic data of 3α-hydroxy-β-cyclocostunolide (**2**)

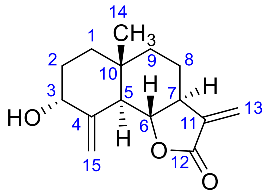

3α-Hydroxy-5,7α(*H*),6β(*H*)-eudesma-4(15),11(13)-dien-6,12-olide, or 3α-hydroxy-β-cyclocostunolide (2). Orangish oil. HRMS (*m/z*) calculated for $C_{15}H_{21}O_3$, 249.1416 $[M - H]^+$; found 249.1485. IR (cm^{-1}): 3460, 1767, 1246, 1126. $[\alpha]^{25}_D = -82°$ ($CHCl_3$, c = 0.5). **^{1}H NMR** (500 MHz, $CDCl_3$, δ, ppm): 1.61 (*m*, 1H, H-1a), 1.46 (*m*, 1H, H-1b), 1.79 (*m*, 1H, H-2a), 1.22 (*m*, 1H, H-2b), 4.29 (*t*, J = 2.3 Hz, 1H, H-3), 2.80 (*d*, J = 11.0 Hz, 1H, H-5), 3.93 (*t*, J = 11.0 Hz, 1H, H-6), 2.59 (*m*, 1H, H-7), 2.02 (*m*, 1H, H-8a), 1.61 (*m*, 1H, H-8b), 1.58 (*m*, 1H, H-9a), 1.48 (*m*, 1H, H-9b), 6.05 (*dd*, J = 0.6, 3.1 Hz, 1H, H-13a), 5.39 (*dd*, J = 0.6, 3.1 Hz, 1H, H-13b), 0.81 (*s*, 3H, H-14), 5.11 (*d*, J = 1.0 Hz, 1H, H-15a), 4.91 (*d*, J = 1.0 Hz, 1H, H-15b). **^{13}C NMR** (125 MHz, $CDCl_3$, δ, ppm): 39.2 (C-1), 28.8 (C-2), 72.7 (C-3), 146.0 (C-4), 48.6 (C-5), 79.8 (C-6), 49.6 (C-7), 21.6 (C-8), 35.8 (C-9), 38.6 (C-10), 139.3 (C-11), 171.0 (C-12), 117.1 (C-13), 17.2 (C-14), 112.0 (C-15).

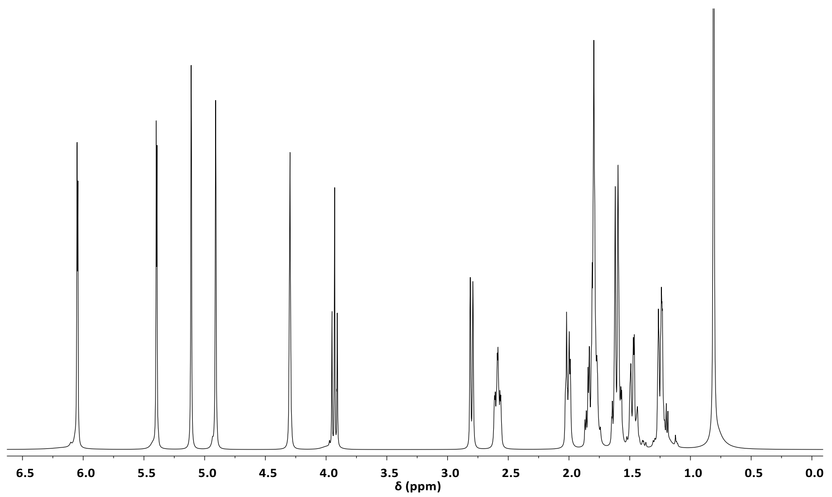

Figure S1.1. ^{1}H NMR spectrum of 3α-hydroxy-β-cyclocostunolide (**2**) in $CDCl_3$ at 500 MHz

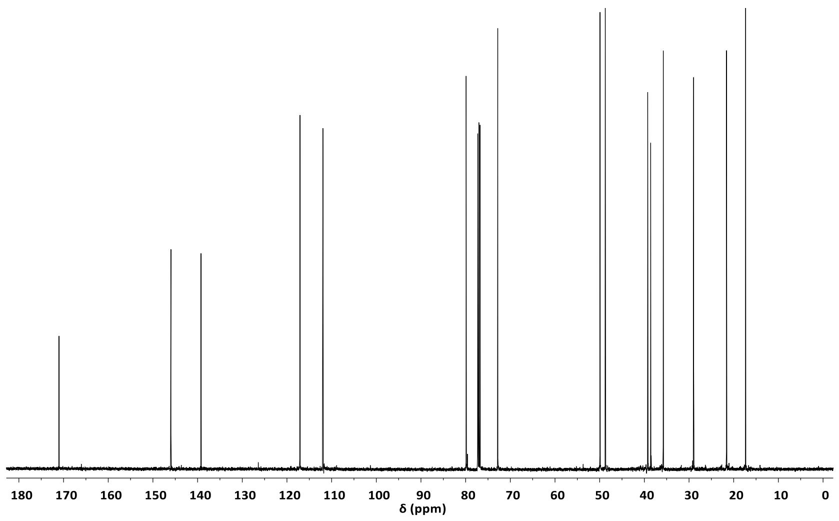

Figure S1.2. ^{13}C NMR spectrum of 3α-hydroxy-β-cyclocostunolide (**2**) in $CDCl_3$ at 125 MHz

S2. Spectroscopic data of 5α-hydroxy-3-deoxybrachylaenolide (**8**)

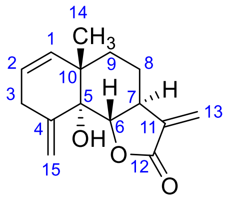

5α-Hydroxy-7α(*H*)-eudesma-1(2),4(15),11(13)-trien-6,12-olide, or 5α-hydroxy-3-deoxybrachylaenolide (8). Yellowish solid, mp 67–69 °C. HRMS (*m/z*) calculated for $C_{15}H_{19}O_3$, 247.1289 $[M - H]^+$; found 247.1329. IR (cm^{-1}): 3488, 1770, 1246, 1136. $[\alpha]^{25}_D$ = +78° ($CHCl_3$, c = 0.05). **^{1}H NMR** (500 MHz, $CDCl_3$, δ, ppm): 6.07 (*dd*, J = 2.9, 12.8 Hz, 1H, H-1), 5.75 (*m*, 1H, H-2), 2.47 (*d*, J = 17.4 Hz, 1H, H-3a), 1.72 (*dd*, J = 5.7, 17.8 Hz, 1H, H-3b), 4.24 (*d*, J = 11.2 Hz, 1H, H-6), 3.31 (*m*, 1H, H-7), 1.99 (*ddd*, J = 2.3, 6.7, 13.2 Hz, 1H, H-8α), 1.61 (*dt*, J = 2.8, 13.5 Hz, 1H, H-8β), 1.42 (*dt*, J = 2.8, 13.5 Hz, 1H, H-9α), 1.82 (*ddd*, J = 4.2, 13.2, 13.2 Hz, 1H, H-9β), 6.11 (*d*, J = 3.3 Hz, 1H, H-13a), 5.42 (*d*, J = 3.3 Hz, 1H, H-13b), 0.93 (*s*, 3H, H-14), 5.56 (*s*, 1H, H-15a), 5.09 (*s*, 1H, H-15b). **^{13}C NMR** (125 MHz, $CDCl_3$, δ, ppm): 127.4/127.5 (C-1 and C-2), 38.2 (C-3), 143.7 (C-4), 74.1 (C-5), 82.7 (C-6), 43.5 (C-7), 21.5 (C-8), 34.3 (C-9), 39.2 (C-10), 139.5 (C-11), 170.6 (C-12), 117.0 (C-13), 21.4 (C-14), 115.2 (C- 15).

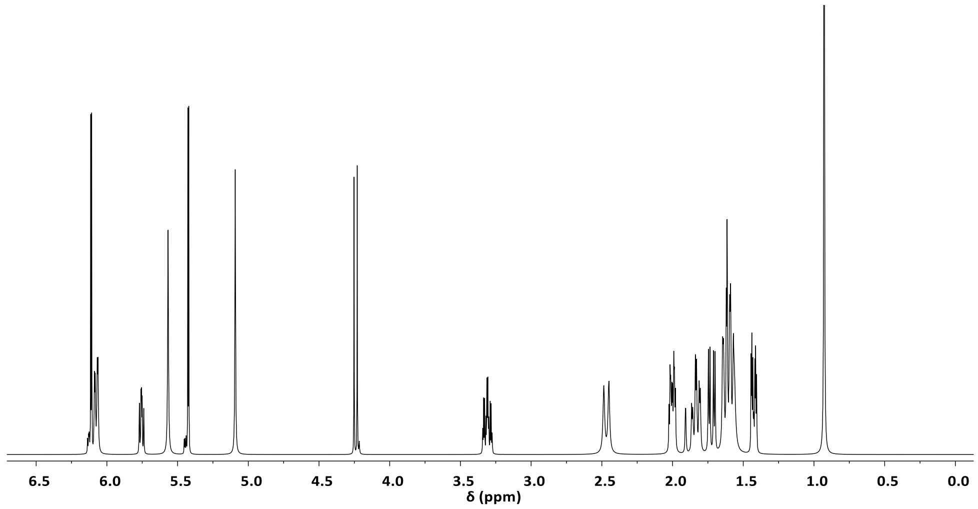

Figure S2.1. ^{1}H NMR spectrum of 3-deoxybrachylaenolide (**8**) in $CDCl_3$ at 500 MHz

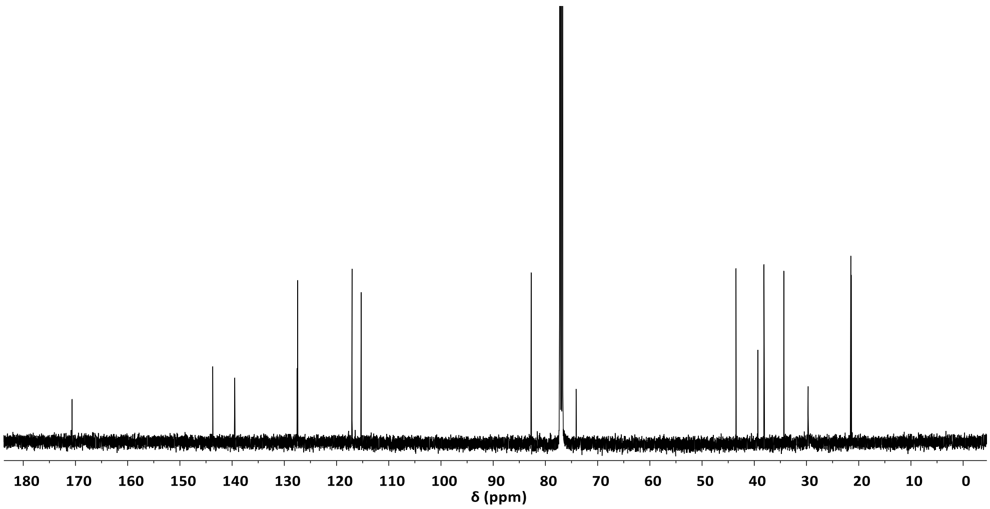

Figure S2.2. ^{13}C NMR spectrum of 3-deoxybrachylaenolide (**8**) in $CDCl_3$ at 125 MHz

S3. Spectroscopic data of 3α,4α-epoxy-5α-hydroxycyclocostunolide (**19**)

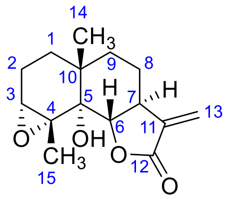

(1a*R*,3a*S*,5a*S*,8a*S*,8b*R*,8c*R*)-8b-hydroxy-3a,8c-dimethyl-6-methylenedecahydrooxireno [2',3':7,8]naphtho[1,2-b]furan-7(2*H*)-one, or 3α,4α-epoxy-5α-hydroxycyclocostunolide (19). Yellowish solid, mp 69–70 °C. HRMS (*m/z*) calculated for $C_{15}H_{20}O_4Na$, 287.1259 $[M-Na]^+$; found 287.1255. IR (cm^{-1}): 2926, 1774, 1247, 1149. $[\alpha]^{25}_D$ = +2.4° ($CHCl_3$, c = 0.48). **^{1}H NMR** (500 MHz, $CDCl_3$, δ, ppm): 1.68 (*ddd*, J = 4.1, 13.1, 13.1 Hz, 1H, H-1a), 1.22 (overlapped signal, 1H, H-1b), 2.10 (br *d*, J = 6.9 Hz, H-2a), 2.06 (br *d*, J = 6.9 Hz, H-2b); 3.20 (*d*, J = 3.2 Hz, H-3), 4.09 (*d*, J = 11.0 Hz, 1H, H-6), 3.31 (br *ddd*, J = 3.3, 3.5, 11.0 Hz, 1H, H-7), 1.96 (*m*, 1H, H-8α), 1.62 (*ddd*, J = 3.8, 12.2, 12.2 Hz, 1H, H-8β), 0.85 (*ddd*, J = 3.2, 13.1, 13.2 Hz, 1H, H-9α), 1.57 (br *dd*, J = 2.6, 12.2 Hz, 1H, H-9β), 6.08 (*d*, J = 3.4 Hz, 1H, H-13a), 5.38 (*d*, J = 3.2 Hz, 1H, H-13b), 0.97 (*s*, 3H, H-14), 1.56 (*s*, 3H, H-15). **^{13}C NMR** (125 MHz, $CDCl_3$, δ, ppm): 33.3 (C-1), 20.7 (C-2), 62.7 (C-3), 61.2 (C-4), 71.6 (C-5), 81.9 (C-6), 43.3 (C-7), 21.2 (C-8), 26.7 (C-9), 38.9 (C-10), 139.8 (C-11), 170.4 (C-12), 116.7 (C-13), 18.5 (C-14), 21.4 (C-15).

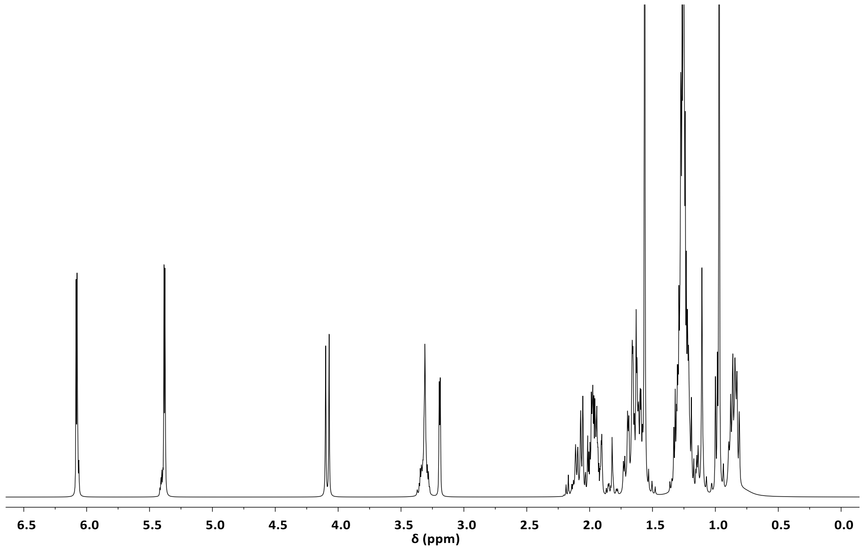

Figure S3.1. ^{1}H NMR spectrum of 3α,4α-epoxy-5α-hydroxycyclocostunolide (**19**) in $CDCl_3$ at 500 MHz

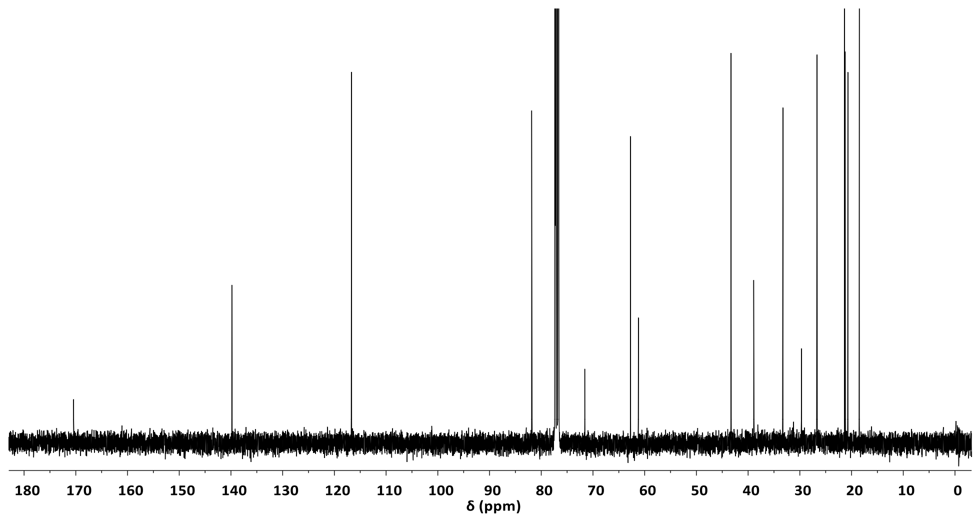

Figure S3.2. ^{13}C NMR spectrum of 3α,4α-epoxy-5α-hydroxycyclocostunolide (**19**) in $CDCl_3$ at 125 MHz

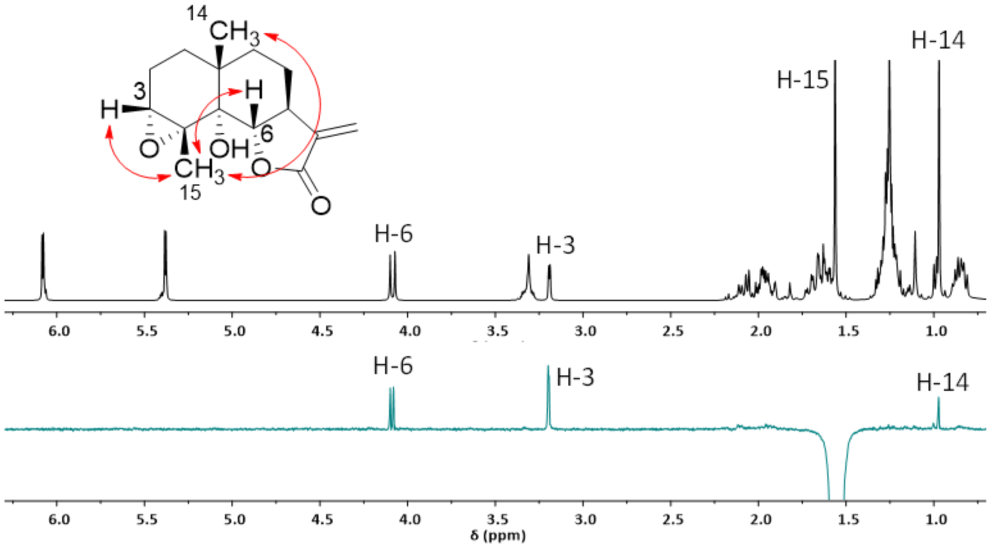

Figure S3.3. NOE 1D spectrum of the H-15 signal (δ 1.56 ppm) of 3α,4α-epoxy-5α-hydroxycyclocostunolide (**19**) in $CDCl_3$ at 500 MHz

S4. Spectroscopic data of 5α-hydroxy-α-cyclocostunolide (**20**)

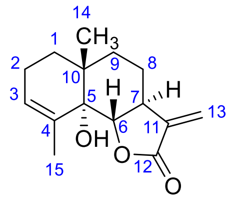

-

(3a*S*,5a*R*,9a*S*,9b*S*)-9a-hydroxy-5a,9-dimethyl-3-methylene-3a,4,5,5a,6,7,9a,9b-octahydronaphtho[1,2-b]furan-2(3*H*)-one, or 5α-hydroxy-α-cyclocostunolide (20). Yellowish oil. HRMS (*m/z*) calculated for $C_{15}H_{21}O_3$, 249.1446 $[M - H]^+$; found 249.1485. IR (cm^{-1}): 3491, 1769, 1141. $[\alpha]^{25}_D$ = +7.8° ($CHCl_3$, c = 0.034). **^{1}H NMR** (500 MHz, $CDCl_3$, δ, ppm): 2.09 (*m*, 2H, H-1), 2.09 (*m*, 1H, H-2a), 1.61 (*m*, 1H, H-2b), 5.51 (*s*, 1H, H-3), 4.08 (*d*, J = 11.0 Hz, 1H, H-6), 3.34 (*m*, 1H, H-7), 1.62 (*m*, 2H, H-8), 1.61 (*m*, 2H, H-9), 6.06 (*d*, J = 3.4 Hz, 1H, H-13a), 5.38 (*d*, J = 3.2 Hz, 1H, H-13b), 0.98 (*s*, 3H, H-14), 1.91 (*dd*, J = 0.6, 2.5, 3H, H-15). **^{13}C NMR** (125 MHz, $CDCl_3$, δ, ppm): 29.7 (C-1), 22.6 (C-2), 126.9 (C-3), 135.6 (C-4), 73.2 (C-5), 82.7 (C-6), 43.2 (C-7), 21.8 (C-8), 33.8 (C-9), 39.2 (C-10), 140.1 (C-11), 170.8 (C-12), 116.4 (C-13), 19.9 (C-14), 14.2 (C-15).

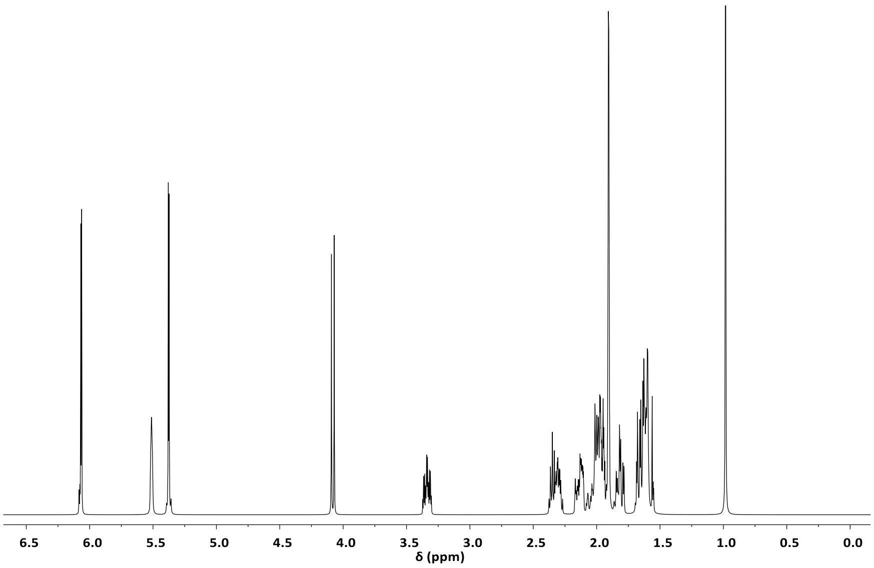

Figure S4.1. ^{1}H NMR spectrum of 5α-hydroxy-α-cyclocostunolide (**20**) in $CDCl_3$ at 500 MHz

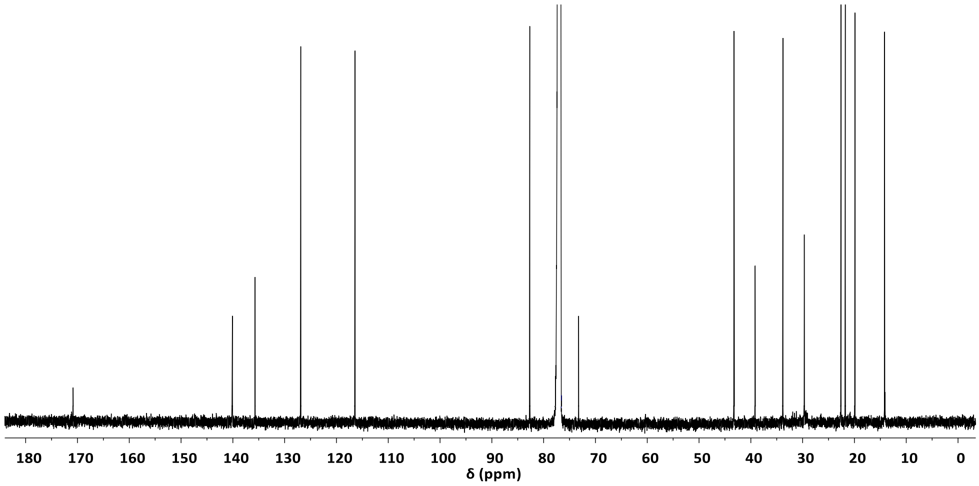

Figure S4.2. ^{13}C NMR spectrum of 5α-hydroxy-α-cyclocostunolide (**20**) in $CDCl_3$ at 125 MHz

S5. Spectroscopic data of 5α,7β-dihydroxy-α-cyclocostunolide (**21**)

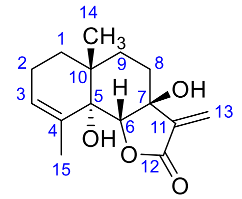

(3a*S*,5a*R*,9a*S*,9b*S*)-3a,9a-dihydroxy-5a,9-dimethyl-3-methylene-3a,4,5,5a,6,7,9a,9b-octahydronaphtho[1,2-b]furan-2(3*H*)-one, or 5α,7β-dihydroxy-α-cyclocostunolide (21). Colorless oil. HRMS (*m/z*) calculated for $C_{15}H_{21}O_4$, 265.1395 $[M - H]^+$; found 265.1434. IR (cm^{-1}): 3490, 1767. $[\alpha]^{25}_D$ = +11° ($CHCl_3$, c = 0.10). **^{1}H NMR** (500 MHz, $CDCl_3$, δ, ppm): 1.45 (*m*, 1H, H-1a), 1.32 (*m*, 1H, H-1b), 2.21 (*m*, 1H, H-2a), 2.06 (*m*, 1H, H-2b), 5.11 (*t*, J = 5.11, 1H, H-3), 5.15 (*s*, 1H, H-6), 1.45 (*m*, 1H, H-8a), 1.28 (*m*, 1H, H-8b), 1.62 (*m*, 2H, H-9), 6.34 (*s*, 1H, H-13a), 5.80 (*s*, 1H, H-13b), 1.10 (*s*, 3H, H-14), 1.79 (*s*, 3H, H-15). **^{13}C NMR** (125 MHz, $CDCl_3$, δ, ppm): 33.5/32.2 (C-1 and C-2), 124.9 (C-3), 129.9 (C-4), 76.9 (C-5), 85.3 (C-6), 76.4 (C-7), 35.1/29.6 (C-8 and C-9), 38.4 (C-10), 141.8 (C-11), 169.1 (C-12), 122.2 (C-13), 25.8 (C-14), 21.5 (C-15).

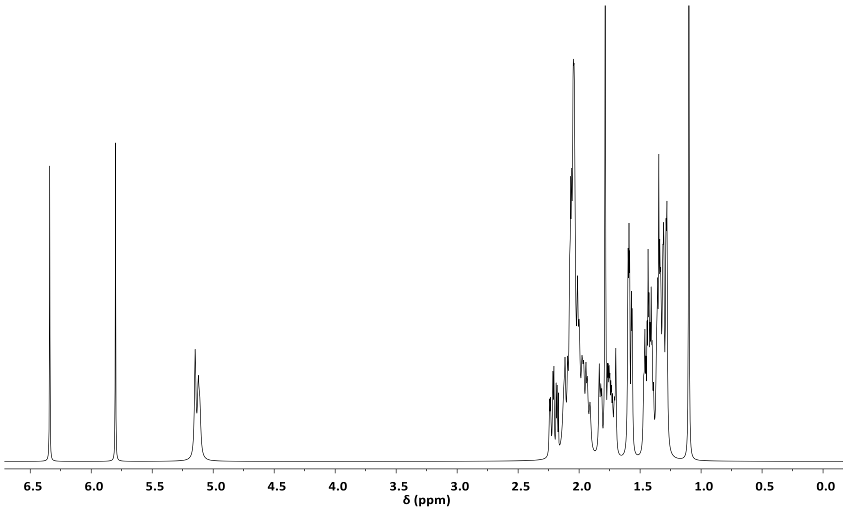

Figure S5.1. ^{1}H NMR spectrum of 5α,7β-dihydroxy-α-cyclocostunolide (**21**) in $CDCl_3$ at 500 MHz

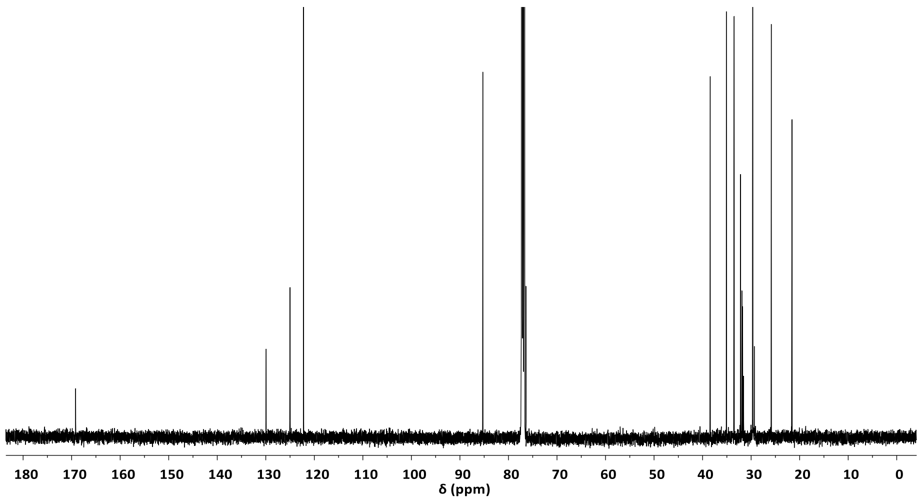

Figure S5.2. ^{13}C NMR spectrum of 5α,7β-dihydroxy-α-cyclocostunolide (**21**) in $CDCl_3$ at 125 MHz

S6. ^{1}H NMR spectrum of costunolide

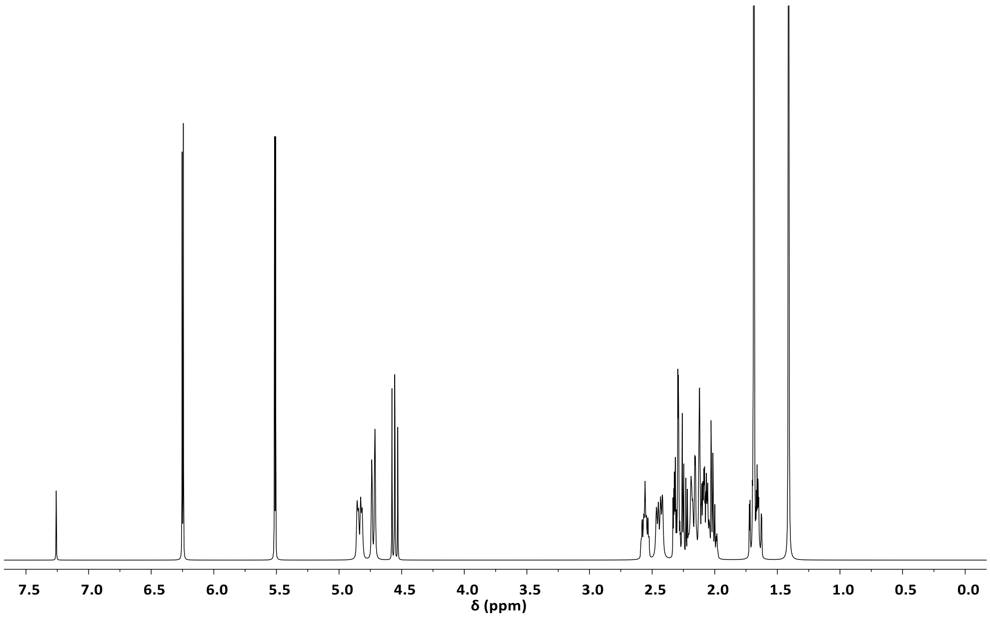

Figure S6. ^{1}H NMR spectrum of costunolide in $CDCl_3$ at 500 MHz

S7. ^{1}H NMR spectra of eudesmanolides (**1**, **3-7** and **9**)

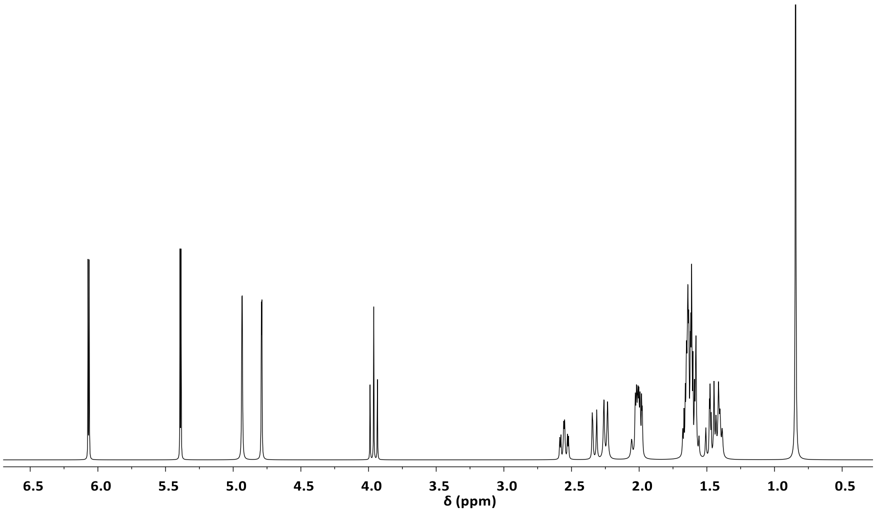

Figure S7.1. ^{1}H NMR spectrum of β-cyclocostunolide (**1**) in $CDCl_3$ at 500 MHz

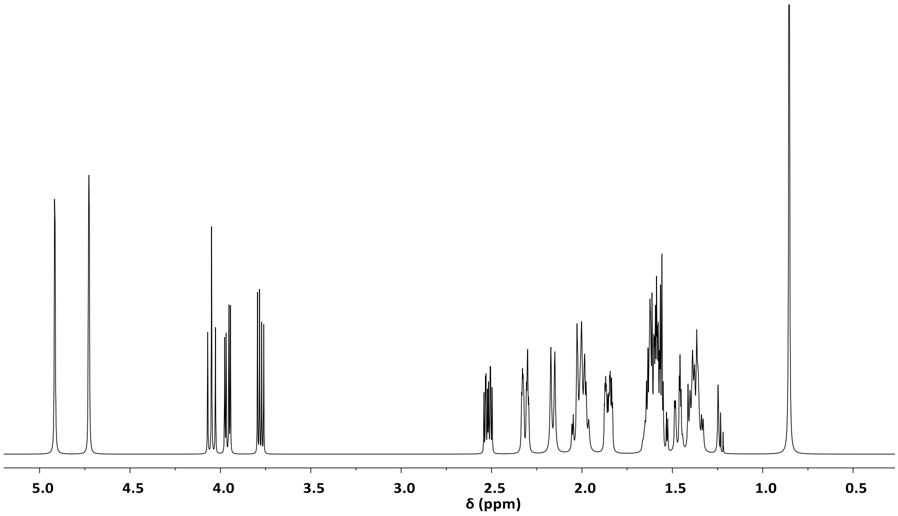

Figure S7.2. ^{1}H NMR spectrum of 11α-hydroxymethyl-β-cyclocostunolide (**3**) in $CDCl_3$ at 500 MHz

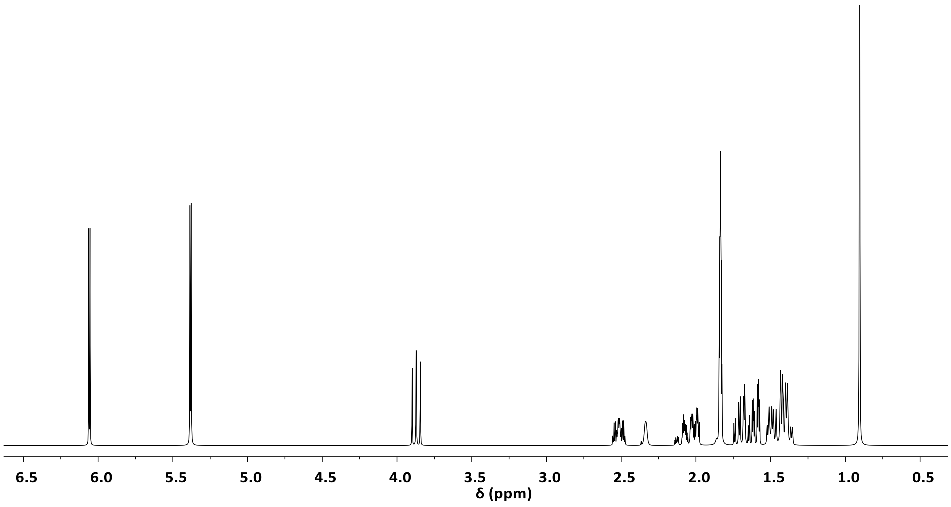

Figure S7.3. ^{1}H NMR spectrum of α-cyclocostunolide (**4**) in $CDCl_3$ at 500 MHz

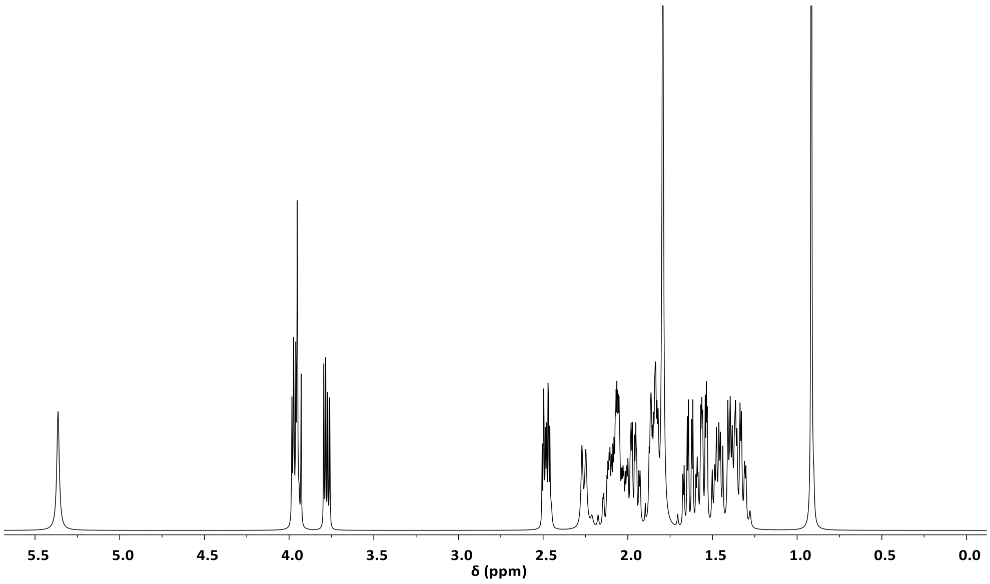

Figure S7.4. ^{1}H NMR spectrum of 11α-hydroxymethyl-α-cyclocostunolide (**5**) in $CDCl_3$ at 500 MHz

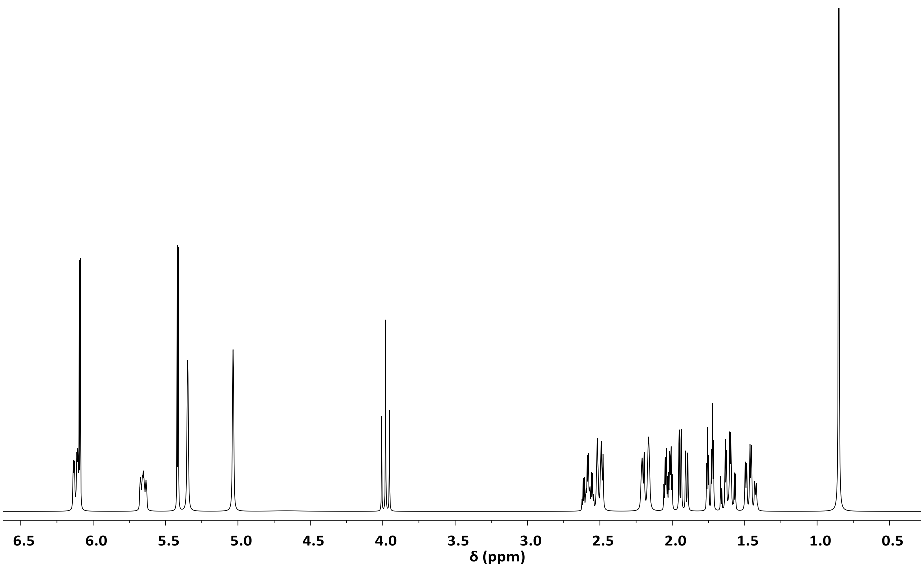

Figure S7.5. ^{1}H NMR spectrum of 3-deoxybrachylaenolide (**6**) in $CDCl_3$ at 500 MHz

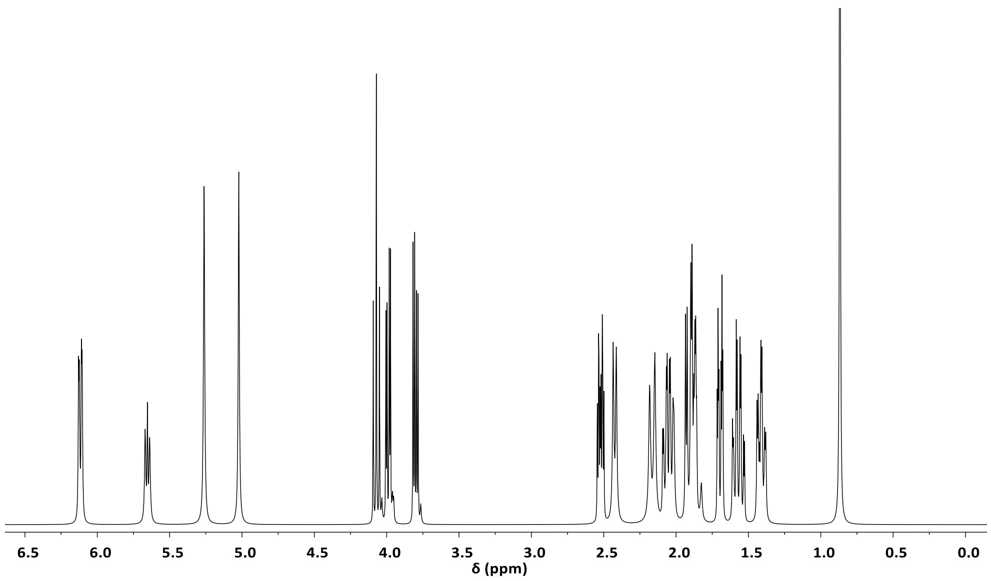

Figure S7.6. ^{1}H NMR spectrum of 11α-hydroxymethyl-3-deoxybrachylaenolide (**7**) in $CDCl_3$ at 500 MHz

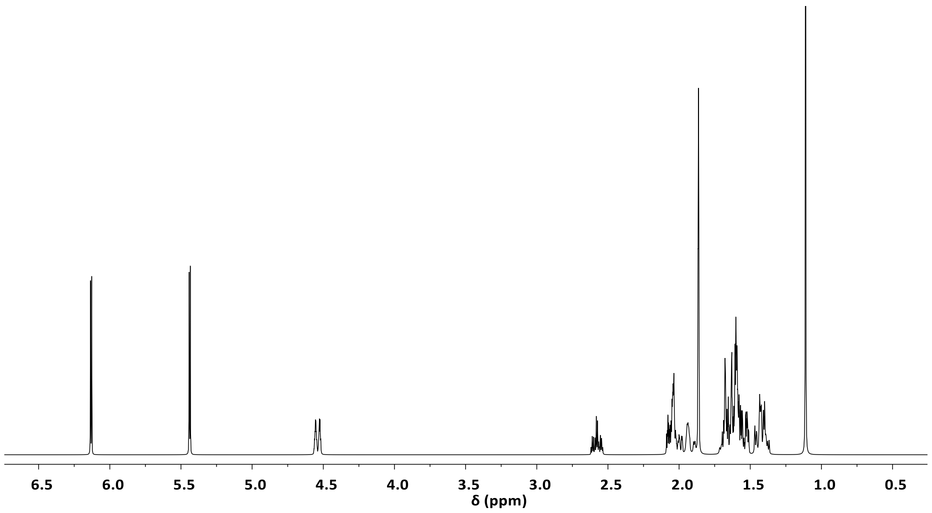

Figure S7.7. ^{1}H NMR spectrum of γ-cyclocostunolide (**9**) in $CDCl_3$ at 500 MHz

S8. ^{1}H NMR spectra of guaianolides (10-18)

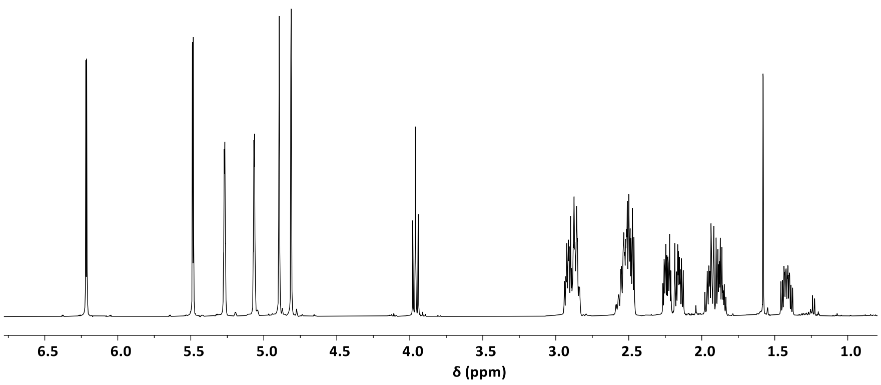

Figure S8.1. ^{1}H NMR spectrum of dehydrocostuslactone (**10**) in $CDCl_3$ at 500 MHz

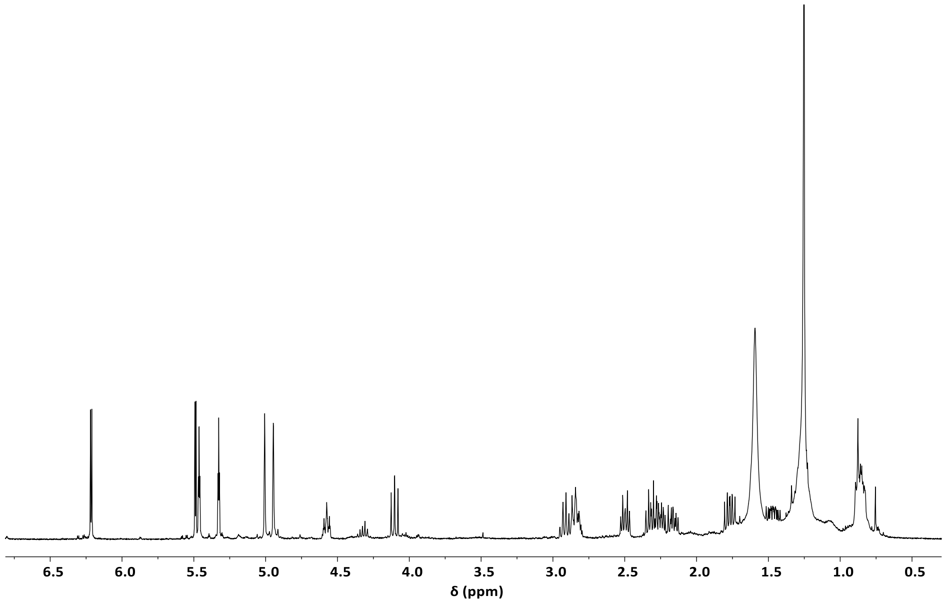

Figure S8.2. ^{1}H NMR spectrum of zaluzanin C (**11**) in $CDCl_3$ at 500 MHz

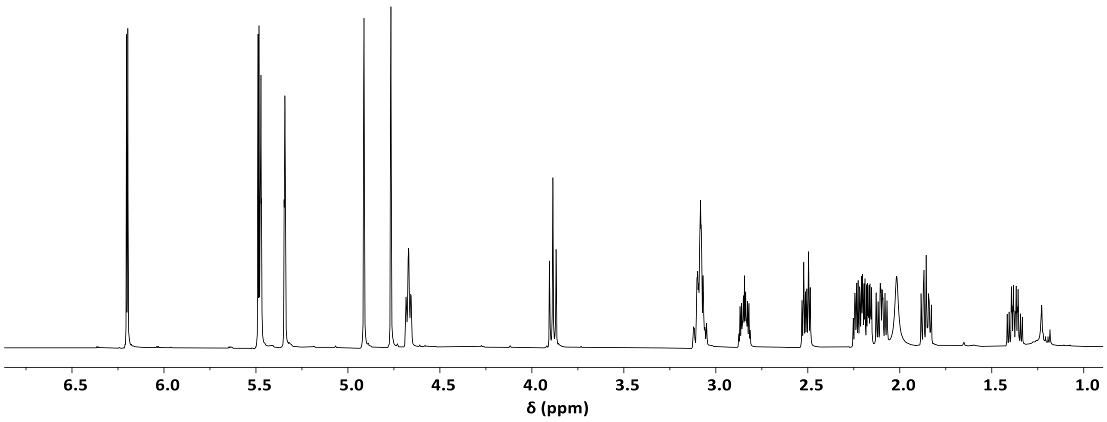

Figure S8.3. ^{1}H NMR spectrum of isozaluzanin C (**12**) in $CDCl_3$ at 500 MHz

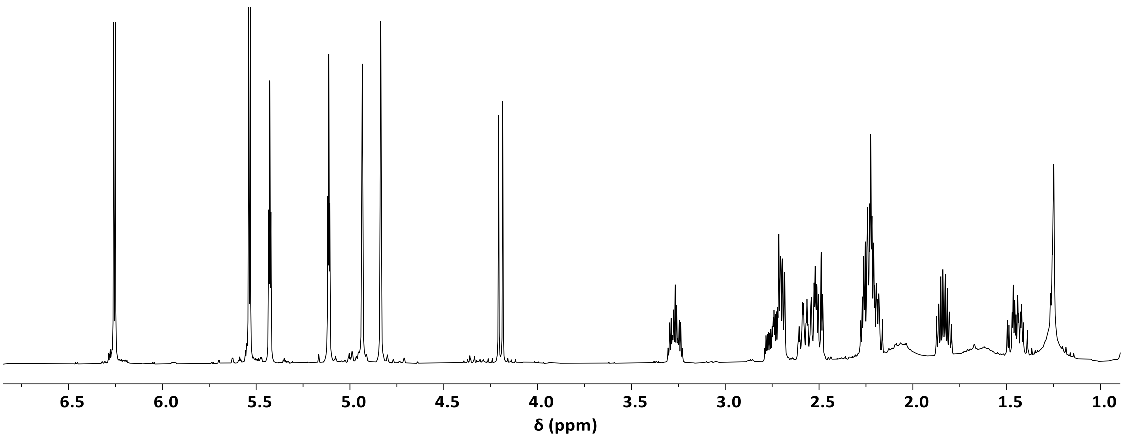

Figure S8.4. ^{1}H NMR spectrum of 5α-hydroxydehydrocostuslactone (**13**) in $CDCl_3$ at 500 MHz

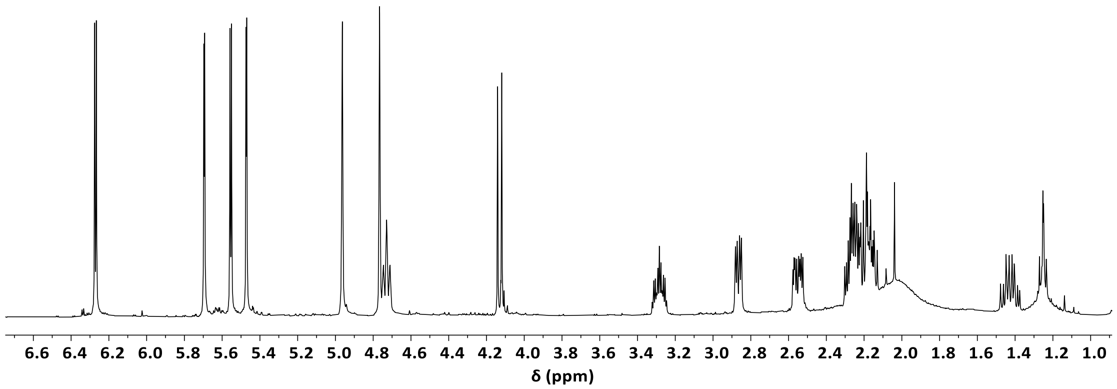

Figure S8.5. ^{1}H NMR spectrum of 5α-hydroxyisozaluzanin C (**14**) in $CDCl_3$ at 500 MHz

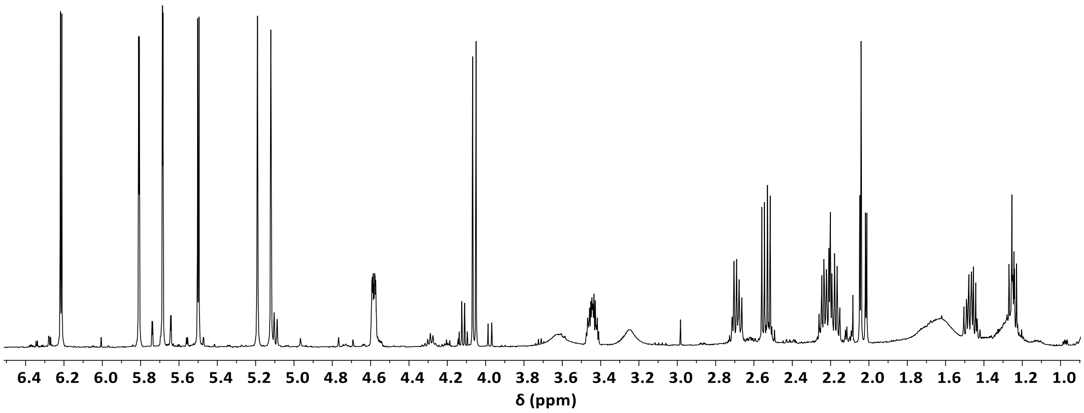

Figure S8.6. ^{1}H NMR spectrum of 1α,5α-dihydroxyisozaluzanin C (**15**) in $CDCl_3$ at 500 MHz

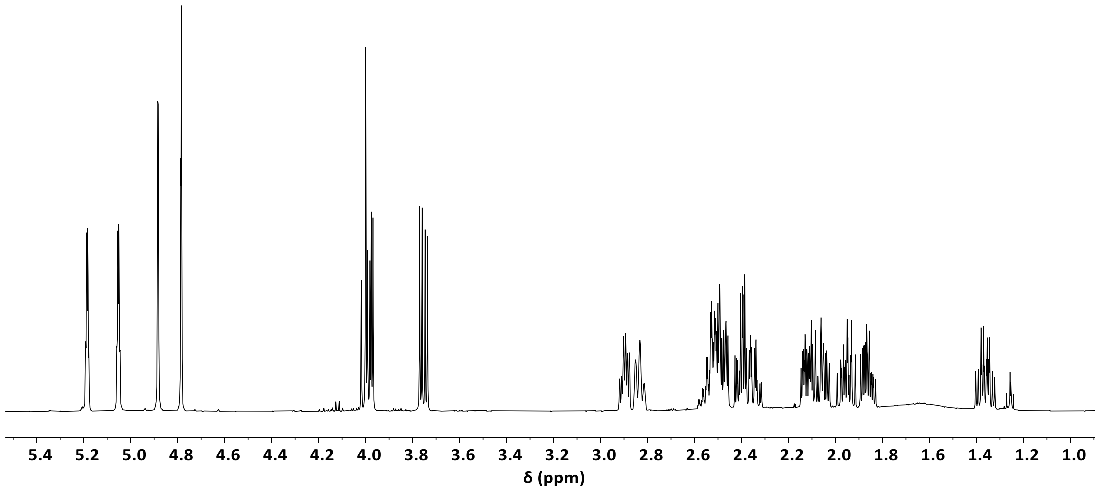

Figure S8.7. ^{1}H NMR spectrum of 11α-hydroxymethyldehydrocostuslactone (**16**) in $CDCl_3$ at 500 MHz

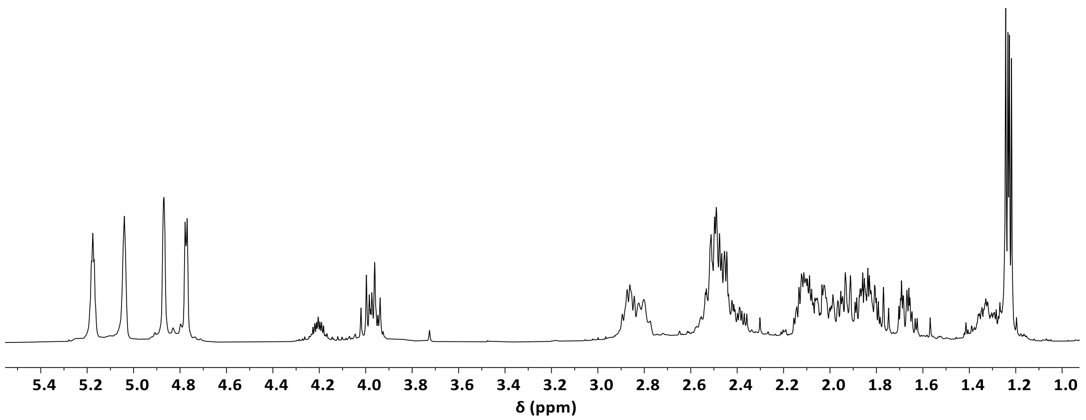

Figure S8.8. ^{1}H NMR spectrum of 11α-(2-hydroxypropyl)dehydrocostuslactone (**17**) in $CDCl_3$ at 500 MHz

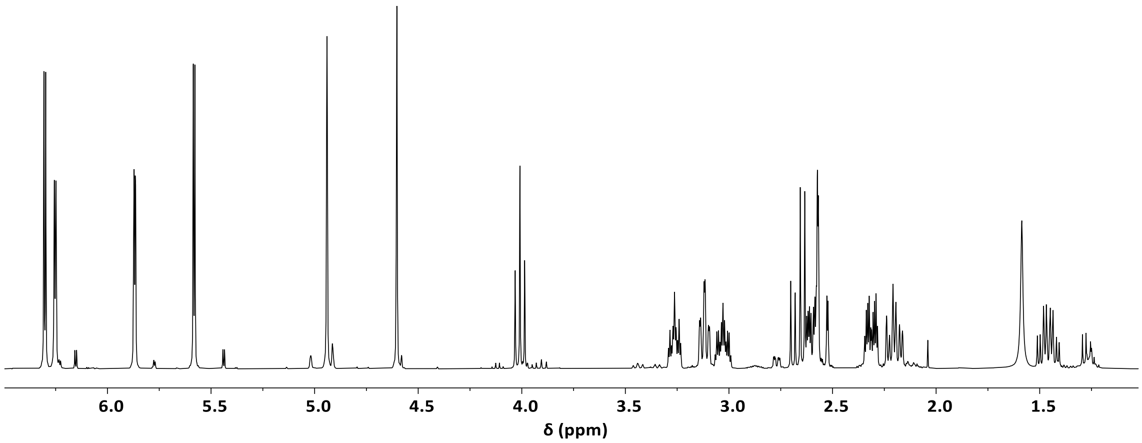

Figure S8.9. ^{1}H NMR spectrum of dehydrozaluzanin C (**18**) in $CDCl_3$ at 500 MHz
